# Neuroradiological, genetic and clinical characteristics of histone H3 K27-mutant diffuse midline gliomas in the Kansai Molecular Diagnosis Network for CNS Tumors (Kansai Network): multicenter retrospective cohort

**DOI:** 10.1186/s40478-024-01808-w

**Published:** 2024-07-27

**Authors:** Nobuhide Hayashi, Junya Fukai, Hirokazu Nakatogawa, Hiroshi Kawaji, Ema Yoshioka, Yoshinori Kodama, Kosuke Nakajo, Takehiro Uda, Kentaro Naito, Noriyuki Kijima, Yoshiko Okita, Naoki Kagawa, Yoshinobu Takahashi, Naoya Hashimoto, Hideyuki Arita, Koji Takano, Daisuke Sakamoto, Tomoko Iida, Yoshiki Arakawa, Takeshi Kawauchi, Yukihiko Sonoda, Yuta Mitobe, Kenichi Ishibashi, Masahide Matsuda, Takamune Achiha, Takahiro Tomita, Masahiro Nonaka, Keijiro Hara, Noriyoshi Takebe, Takashi Tsuzuki, Yoshikazu Nakajima, Shiro Ohue, Nobuyuki Nakajima, Akira Watanabe, Akihiro Inoue, Masao Umegaki, Daisuke Kanematsu, Asako Katsuma, Miho Sumida, Tomoko Shofuda, Masayuki Mano, Manabu Kinoshita, Kanji Mori, Naoyuki Nakao, Yonehiro Kanemura

**Affiliations:** 1 Department of Neurosurgery, Wakayama Rosai Hospital, Kinomoto 93-1, Wakayama City, Wakayama 640-8505 Japan; 2 Kansai Molecular Diagnosis Network for CNS Tumors, Osaka City, Osaka 540-0006 Japan; 3 Department of Neurological Surgery, School of Medicine, Wakayama Medical University, Kimiidera 811-1, Wakayama City, Wakayama 641-8510 Japan; 4 Department of Pediatric Neurosurgery, Seirei Hamamatsu General Hospital, Hamamatsu, Shizuoka 430-8558 Japan; 5 Department of Neurosurgery, Seirei Hamamatsu General Hospital, Hamamatsu, Shizuoka 430-8558 Japan; 6 Division of Molecular Medicine, Department of Biomedical Research and Innovation, Institute for Clinical Research, NHO Osaka National Hospital, Osaka City, Osaka 540-0006 Japan; 7 Department of Diagnostic Pathology and Cytology, Osaka International Cancer Institute, Osaka City, Osaka 541-8567 Japan; 8 Department of Neurosurgery, Osaka Metropolitan University Graduate School of Medicine, Osaka City, Osaka 545-8585 Japan; 9 Department of Neurosurgery, Osaka University Graduate School of Medicine, Suita, Osaka 565-0871 Japan; 10 Department of Neurosurgery, School of Medical Science, Kyoto Prefectural University Graduate, Kyoto City, Kyoto 602-8566 Japan; 11 Department of Neurosurgery, Osaka International Cancer Institute, Osaka City, Osaka 541-8567 Japan; 12 Department of Neurosurgery, Hyogo College of Medicine, Nishinomiya, Hyogo 663-8501 Japan; 13 Department of Neurosurgery, Kyoto University Graduate School of Medicine, Kyoto City, Kyoto 606-8507 Japan; 14 Department of Neurosurgery, Osaka Red Cross Hospital, Osaka City, Osaka 543-8555 Japan; 15 Department of Neurosurgery, Faculty of Medicine, Yamagata University, Yamagata City, Yamagata 990-8560 Japan; 16 Department of Neurosurgery, Osaka City General Hospital, Osaka City, Osaka, 534-0021 Japan; 17 Department of Neurosurgery, Faculty of Medicine, University of Tsukuba, Tsukuba, Ibaraki 305-8575 Japan; 18 Department of Neurosurgery, Kansai Rosai Hospital, Amagasaki, Hyogo 660-8511 Japan; 19 Department of Neurosurgery, Graduate School of Medicine and Pharmaceutical Sciences, University of Toyama, Toyama City, Toyama, 930-0194 Japan; 20 Department of Neurosurgery, Kansai Medical University, Hirakata, Osaka 573-1191 Japan; 21 Department of Neurosurgery, Tokushima University Graduate School of Biomedical Sciences, Tokushima City, Tokushima, 770-8501 Japan; 22 Department of Neurosurgery, Medical Research Institute, Tazuke Kofukai Foundation, Kitano Hospital, Osaka City, Osaka, 530-8480 Japan; 23 Department of Neurosurgery, Sakai City Medical Center, Sakai, Osaka 593-8304 Japan; 24 Department of Neurosurgery, Kobe Tokushukai Hospital, Kobe, Hyogo 655-0017 Japan; 25 Department of Neurosurgery, Ehime Prefectural Central Hospital, Matsuyama, Ehime 790-0024 Japan; 26 Department of Neurosurgery, Tokyo Medical University, Tokyo, 160-0023 Japan; 27 Department of Neurosurgery, Kindai University Nara Hospital, Ikoma, Nara 630-0293 Japan; 28 Department of Neurosurgery, Ehime University School of Medicine, Toon, Ehime 791-0295 Japan; 29 Department of Neurosurgery, Suita Municipal Hospital, Suita, Osaka 564-8567 Japan; 30 Division of Regenerative Medicine, Department of Biomedical Research and Innovation, Institute for Clinical Research, NHO Osaka National Hospital, Osaka City, Osaka, 540-0006 Japan; 31 Division of Stem Cell Research, Department of Biomedical Research and Innovation, Institute for Clinical Research, NHO Osaka National Hospital, Osaka City, Osaka, 540-0006 Japan; 32 Department of Central Laboratory and Surgical Pathology, NHO Osaka National Hospital, Osaka City, Osaka, 540-0006 Japan; 33 Department of Neurosurgery, Asahikawa Medical University, Asahikawa, Hokkaido 078-8510 Japan; 34 Department of Neurosurgery, Yao Municipal Hospital, Yao, Osaka 581-0069 Japan; 35 Department of Neurosurgery, NHO Osaka National Hospital, Osaka City, Osaka, 540-0006 Japan

**Keywords:** Diffuse midline glioma, H3 K27-altered, Midline location, Clinical characteristic, Molecular feature, Survival, Prognostic factor

## Abstract

This study aims to elucidate the clinical and molecular characteristics, treatment outcomes and prognostic factors of patients with histone H3 K27-mutant diffuse midline glioma. We retrospectively analyzed 93 patients with diffuse midline glioma (47 thalamus, 24 brainstem, 12 spinal cord and 10 other midline locations) treated at 24 affiliated hospitals in the Kansai Molecular Diagnosis Network for CNS Tumors. Considering the term “midline” areas, which had been confused in previous reports, we classified four midline locations based on previous reports and anatomical findings. Clinical and molecular characteristics of the study cohort included: age 4–78 years, female sex (41%), lower-grade histology (56%), preoperative Karnofsky performance status (KPS) scores ≥ 80 (49%), resection (36%), adjuvant radiation plus chemotherapy (83%), temozolomide therapy (76%), bevacizumab therapy (42%), *HIST1H3B* p.K27M mutation (2%), *TERT* promoter mutation (3%), *MGMT* promoter methylation (9%), *BRAF* p.V600E mutation (1%), *FGFR1* mutation (14%) and *EGFR* mutation (3%)*.* Median progression-free and overall survival time was 9.9 ± 1.0 (7.9–11.9, 95% CI) and 16.6 ± 1.4 (13.9–19.3, 95% CI) months, respectively. Female sex, preoperative KPS score ≥ 80, adjuvant radiation + temozolomide and radiation ≥ 50 Gy were associated with favorable prognosis. Female sex and preoperative KPS score ≥ 80 were identified as independent good prognostic factors. This study demonstrated the current state of clinical practice for patients with diffuse midline glioma and molecular analyses of diffuse midline glioma in real-world settings. Further investigation in a larger population would contribute to better understanding of the pathology of diffuse midline glioma.

**Supplementary Information:**

The online version contains supplementary material available at 10.1186/s40478-024-01808-w.

## Introduction

Diffuse midline glioma (DMG) harboring histone H3 K27 mutation is diagnosed as DMG, H3 K27-altered in World Health Organization Classification of Tumors of the Central Nervous System 2021 (CNS WHO 2021). It is characterized by the loss of histone H3 p.K28me3 (K27me3), which contains the H3 c.83A > T p.K28M(K27M) substitution in H3.3 (*H3F3A*) or H3.1 (*HIST1H3B/C*) [57]. DMG is categorized as a pediatric-type diffuse high-grade glioma in CNS WHO 2021 [57]. However, DMG may occur in adults as well as in children and adolescents, and this has created confusion over the diagnosis and treatment of adult diffuse gliomas, with differing definitions being used [14, 24, 26, 27, 33, 35, 42–44, 51, 55, 56, 60–62].

Essential information about DMG has been summarized in the WHO Blue Book [57]. Even after CNS WHO 2021, however, several researchers have reported additional findings [5, 6, 23, 27, 30, 32, 36, 53, 54, 58, 62]. Owing to its rarity, however, there are few comprehensive reports and there are remaining inconsistencies about DMG. There are major concerns regarding prediction of clinical behavior and outcomes in daily practice; there is a lack of real-world data on clinical and molecular characteristics and treatment outcomes. The current study investigates the prevalence and impact of previously-reported biomarkers.

DMG is defined as tumors located in areas such as the thalamus, the brainstem and the spinal cord, and occasionally in the pineal gland, the hypothalamus, and the cerebellum [57]. On the other hand, H3 K27M mutation has reportedly been detected in not only in tumors of these areas, but also in those of other locations, such as in the cerebral hemisphere, the corpus callosum, the ventricles, the basal ganglia, and the suprasellar region [1, 2, 7, 11, 14, 17, 19, 20, 23, 25, 31, 33, 39, 42, 47, 51, 55, 62] (Additional file 1: Table S1). As for the basal ganglia and corpus callosum, some researchers have regarded them as “midline” structures [1, 2, 7, 23, 25, 31, 39, 42, 51, 55, 62], while others have regarded them as “non-midline” structures and these tumors have thus been excluded from DMG [11, 19, 40, 60] (Additional file 1: Table S1). Discrimination between the “midline” and “non-midline” structures for definition of DMG therefore lacks consensus.

For the present study, we reviewed the inclusion criteria of DMG used in previous reports that focused upon the midline structures. We collected histone H3 K27M-mutant diffuse gliomas at the midline location in the Kansai Molecular Diagnosis Network for CNS Tumors (Kansai Network) cohort. This is a multi-institutional retrospective cohort study of 93 cases of DMG treated at 24 hospitals in the Kansai Network. We aim to elucidate both clinical and pathological features of cases of DMG, as well as treatment outcomes and prognostic factors of patients with DMG in real-world settings.

## Material and methods

### Ethics

This study was carried out in accordance with the principles of the Declaration of Helsinki. The study was approved by the Institutional Review Board (IRB) of Osaka National Hospital (No. 713), Wakayama Medical University (No. 98), Wakayama Rosai Hospital (No. 20 Res-17), and all collaborating institutions. Written informed consent was obtained from all patients.

### Patient population and study design

**Fig. 1:**
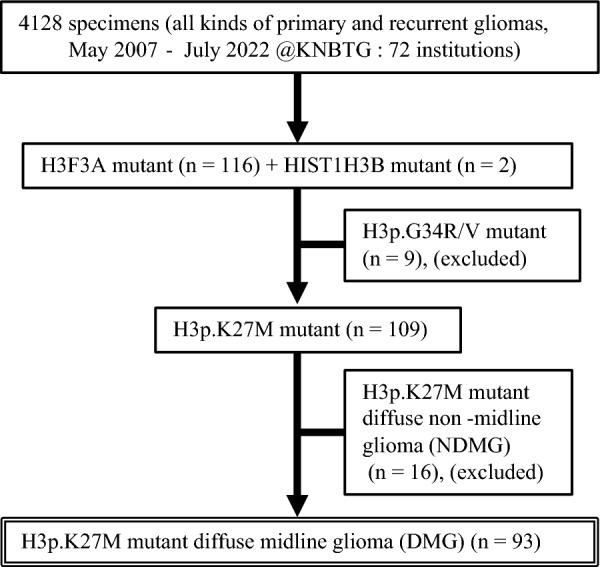
Flowchart of patient selection

This study included patients with histone H3-mutated gliomas who were treated at one of 27 institutions or hospitals participating in the Kansai Network [41]. Between May 2007 and July 2022, we collected a total of 4128 samples including all kinds of primary and recurrent gliomas from 72 institutions. From this databank, we focused on diffuse gliomas with histone H3 mutation and collected 118 cases (116 cases with *H3F3A* mutation and two cases with *HIST1H3B* mutation). Among the cases with *H3F3A* mutation, 107 cases had the *K27M* mutation, and nine cases had the *G34R/V* mutation. In this study, we examined 109 cases from 24 institutions, consisting of 107 cases with the *K27M* mutation and two cases with the *HIST1H3B* mutation. Patient selection is summarized in a flowchart in Fig. 1. Diagnosis of diffuse gliomas was initially confirmed by histopathological examination at each institution or hospital.

### Tumor location (Kansai classification)

Preoperative images were available in 106 of the 109 cases (the anatomic tumor locations were identified by medical records in three cases). Neuroradiological assessments were performed by three experienced board-certified neurosurgeons (N.H., H.N., H.K.) and three additional senior board-certified neurosurgeons (J.F., K.M., Yo.Ka.) to reach a consensus. Tumor locations in this study were determined using the anatomical criteria as follows:

- The main anatomical structure in which the tumor is solely located is defined as the tumor location, for example, the thalamus, the brainstem, the spinal cord, etc. (Additional file 4: Figure S1A).
- If tumors were distributed across multiple anatomical regions in a contiguous manner, the presumed tumor origin site was determined based on the location of contrast-enhanced lesions and the progression pattern of FLAIR high-signal areas (Additional file 4: Figure S1B).
- The cases in which non-contiguous multifocal tumors were detected and in which the main anatomical structure of the tumor could not be determined were defined as unclassified. For example, a case might equally harbor both the thalamus and the corpus callosum (Additional file 4: Figure S1C, D).

To discriminate between the “midline” and “non-midline” locations for this study, we applied the following criteria:

- The thalamus, brainstem, spinal cord, pineal gland, hypothalamus, cerebellum, and ventricles were categorized as midline, and the basal ganglia and corpus callosum (as part of the cerebral hemisphere) were categorized as non-midline [45, 49, 50] (Table 1).
- If a tumor was located at the basal ganglia or corpus callosum but mainly involved midline structures such as the thalamus or the brainstem, it was categorized as a midline tumor (Additional file 4: Figure S1C, E, Table 1).
- If a tumor mainly involved the cerebral hemisphere, it was categorized as a non-midline tumor (Additional file 4: Figure S1F, Table 1).

**Table 1:** Kansai classification by site of histone H3 K27M mutant diffuse glioma (n = 109)

DMG; "midline" (n = 93)	NDMG; "non-midline" (n = 16)
Thalamus	Cerebral hemisphere
Brainstem	Corpus callosum
Spinal cord	Basal ganglia
Pineal body	
Subthalamus	
Cerebellum	
Ventricle	
Unclassified + mainly midline structures*	Unclassified + mainly cerebral hemisphere*
Corpus callosum + mainly midline structures^†^	Corpus callosum + mainly cerebral hemisphere^†^
Basal ganglia + mainly midline structures^‡^	Basal ganglia + mainly cerebral hemisphere^‡^

*The main location could not be determined

^†^^,‡^Distinguished based on dominance of either the midline structures or cerebral hemisphere

### Clinical information

Clinical information was collected from medical records including patient demographics, preoperative Karnofsky performance status (KPS) scores, the extent of surgical resection (EOR), adjuvant radiation and chemotherapy regimens, and survival time. EOR was classified according to the assessment by the surgeon as either gross total resection (GTR, 100% of the tumor was resected), subtotal resection (STR, 80–99%), partial resection (PR, < 80%), or biopsy. Patients either received no adjuvant treatment regimen, or those consisting of radiation (RT) plus chemotherapy, RT alone, or chemotherapy alone. Chemo-agents included temozolomide (TMZ), nimustine hydrochloride (ACNU), and bevacizumab (BEV). Adjuvant treatment regimens were determined by the attending physicians’ consideration of the patient’s condition.

### Histopathological examination

All cases were subject to central pathology review by a senior board-certified neuropathologist (Yo.Ko). Histological diagnosis was made based on the CNS WHO 2021 classification [57].

### Genetic analysis

Frozen or fresh tumor samples were obtained during surgery, and tumor genomic DNA was extracted from those tissues for genetic analysis [41]. Briefly, the methylation status of *MGMT* promoter (*MGMT*p) was analyzed by quantitative methylation-specific PCR after bisulfite modification of genomic DNA, and a threshold of ≥ 1% was used for *MGMT*p methylation. The presence of hotspot mutations in *H3F3A, HIST1H3B, IDH1* (R132), *IDH2* (R172), *TERT* promoter, *BRAF* (V600), *FGFR1* (exon12 and exon14) and *EGFR* (exon 7 and exon20) genes, and all exons of *TP53* were analyzed by Sanger sequencing [4, 52, 58].

### Statistical analysis

Statistical analysis was performed using the SAS package and JMP Pro version 16 (SAS Institute, Cary, NC, USA) and the SPSS Statistics version 29 (IBM, NY, USA, 2022). Categorized data were compared between subgroups using the Kruskal–Wallis test (age: continuous factor) and Pearson’s chi-square test (other items: nominal scale). Progression-free survival (PFS) and overall survival (OS) curves were estimated by the Kaplan–Meier method and compared with the log-rank test. Multivariate analyses of prognostic factors were performed using the Cox proportional hazards model. A *p* value of < 0.05 was considered statistically significant.

## Results

Preoperative imaging analysis resulted in 93 of 109 cases being categorized as having midline tumors (diffuse midline tumor, DMG) (85%) and they were enrolled in this study. The other sixteen cases (15%) were categorized as having non-midline tumors.

**Fig. 2:**
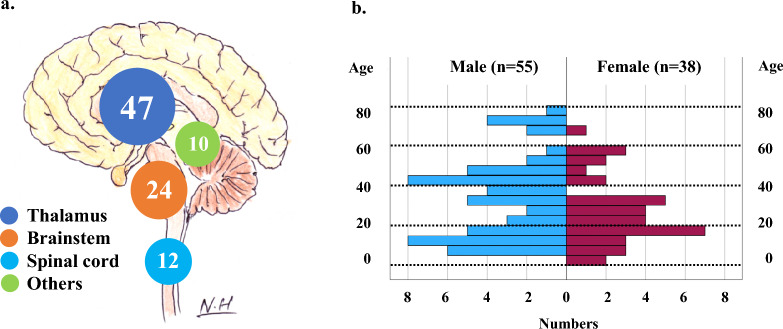
**a** Schematic illustration of tumor locations and each number. Thalamus: 47, Brainstem: 24, Spinal cord: 12, Others: 10. **b** Distribution of patients’ age and sex. There are 26 cases (≤ 18 years) (28%), 60 cases (64%) (19–69 years) and 7 cases (≥ 70 years) (8%)

**Fig. 3:**
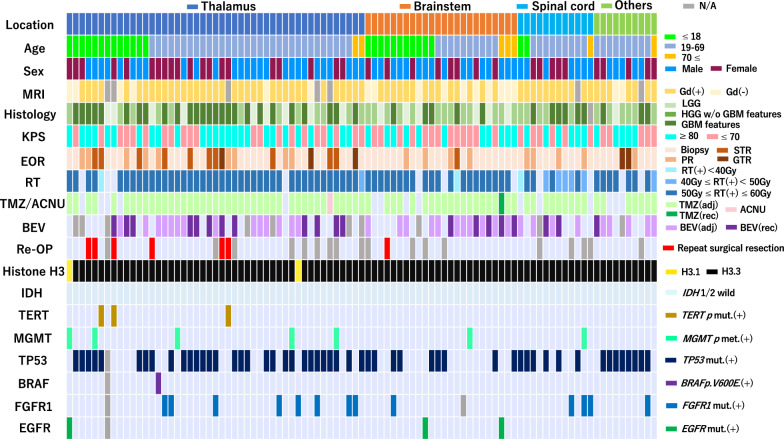
Tile panel demonstrating clinical and molecular characteristics of histone H3 K27-mutant diffuse midline glioma patients in Kansai Network (n = 93)

The clinical and molecular characteristics of the 93 patients analyzed in this study are shown in Table 2. Anatomical tumor locations were classified into four groups: the thalamus group (47 cases), the brainstem group (24 cases), the spinal cord group (12 cases) and other midline locations group (10 cases) (Fig. 2a, Table 2). Other midline locations included the ventricle (two cases), the basal ganglia (two cases), and the cerebellum (2 cases), and four cases were unclassified. Cases in the basal ganglia and unclassified cases mainly involved midline locations. Distribution of the patients’ age and sex are shown in Fig. 2b, and detailed information on each patient is shown as a tile panel in Fig. 3.

**Table 2:** Clinical and molecular characteristics of histone H3 K27-mutant diffuse midline glioma patients in Kansai Network (n = 93)

		Total	Location				
			Thalamus	Brainstem	Spinal cord	Others	*p* value
Number		93	47 (51%)	24 (26%)	12 (13%)	10 (11%)	
Clinical characteristics							
Age (years)	Median (range)	31 (4–78)	28 (4–76)	21 (6–75)	30 (12–78)	45 (36–71)	0.041*
Sex							0.809
	Male	55 (59%)	27 (57%)	16 (67%)	7 (58%)	5 (50%)	
	Female	38 (41%)	20 (43%)	8 (33%)	5 (42%)	5 (50%)	
MR images (Gd enhancement)							0.016*
	High grade features	68 (73%)	36 (76%)	14 (58%)	11 (92%)	7 (70%)	
	Low grade featrues	18 (19%)	6 (13%)	10 (42%)	0 (–)	2 (20%)	
	Unknown	7 (8%)	5 (11%)	0 (–)	1 (8%)	1 (10%)	
Histopathology (CNS WHO 2021)							0.019*
	LGG	16 (17%)	9 (19%)	6 (25%)	0 (–)	1 (10%)	
	HGG without GBM features	36 (39%)	12 (26%)	13 (54%)	5 (42%)	6 (60%)	
	GBM features	40 (43%)	26 (55%)	5 (21%)	6 (50%)	3 (30%)	
	Unknown	1 (1%)	0 (–)	0 (–)	1 (8%)	0 (–)	
Preoperative KPS score							0.568
	80–100	46 (49%)	26 (55%)	11 (46%)	4 (33%)	5 (50%)	
	-70	47 (51%)	21 (45%)	13 (54%)	8 (67%)	5 (50%)	
Extent of surgical resection (EOR)							0.05
	GTR	5 (5%)	3 (6%)	0 (–)	0 (–)	2 (20%)	
	STR	11 (12%)	10 (21%)	1 (4%)	0 (–)	0 (–)	
	PR	18 (19%)	9 (19%)	4 (17%)	4 (33%)	1 (10%)	
	Biopsy	59 (63%)	25 (53%)	19 (79%)	8 (67%)	7 (70%)	
Adjuvant treatment							0.588
	RT + TMZ + BEV	35 (38%)	17 (36%)	11 (46%)	3 (25%)	4 (40%)	
	RT + TMZ	37 (40%)	23 (49%)	6 (25%)	6 (50%)	2 (20%)	
	RT + ACNU	1 (1%)	1 (2%)	0 (–)	0 (–)	0 (–)	
	RT + BEV	4 (4%)	1 (2%)	1 (4%)	1 (8%)	1 (10%)	
	RT alone	5 (5%)	1 (2%)	3 (13%)	0 (–)	1 (10%)	
	TMZ alone	4 (4%)	1 (2%)	2 (8%)	0 (–)	1 (10%)	
	BEV alone	0 (–)	0 (–)	0 (–)	0 (–)	0 (–)	
	None	7 (8%)	3 (6%)	1 (4%)	2 (17%)	1 (10%)	
Radiation dose (Gy)							< 0.001*
	50–60	74 (80%)	41 (87%)	20 (83%)	4 (33%)	7 (70%)	
	40–49	8 (9%)	1 (2%)	0 (–)	5 (42%)	1 (10%)	
	< 40 (range 35–39)	4 (4%)	1 (2%)	1 (4%)	1 (8%)	0 (–)	
	None	7 (8%)	4 (9%)	3 (13%)	2 (17%)	2 (20%)	
Bevacizumab	(Adjuvant + Recurrent)	53 (57%)	25 (53%)	16 (67%)	7 (58%)	5 (50%)	0.943
Repeat surgical resection		7 (12%)	6 (20%)	1 (7%)	0 (–)	0 (–)	0.398
Genetic status							
Histone mutation							0.572
	HIST1H3B p.K27M	2 (2%)	2 (4%)	0 (–)	0 (–)	0 (–)	
	H3F3A p.K27M	91 (98%)	45 (96%)	24 (100%)	12 (100%)	10 (100%)	
*IDH1/2*							–
	Wild	93 (100%)	47 (100%)	24 (100%)	12 (100%)	10 (100%)	
	Mut	0 (–)	0 (–)	0 (–)	0 (–)	0 (–)	
*TERT* promoter							0.386
	Wild	90 (97%)	44 (94%)	24 (100%)	12 (100%)	10 (100%)	
	C228T/C250T	3 (3%)	3 (6%)	0 (–)	0 (–)	0 (–)	
*MGMT* promoter							0.304
	Methylated	8 (9%)	5 (11%)	1 (4%)	0 (–)	2 (20%)	
	Unmethylated	85 (91%)	42 (89%)	23 (96%)	12 (100%)	8 (80%)	
*TP53*							0.207
	Wild	39 (42%)	16 (34%)	15 (63%)	6 (50%)	2 (20%)	
	Mutation	53 (57%)	30 (64%)	9 (38%)	6 (50%)	8 (80%)	
	Unknown	1 (1%)	1 (2%)	0 (–)	0 (–)	0 (–)	
*BRAF*							0.92
	Wild	91 (98%)	45 (96%)	24 (100%)	12 (100%)	10 (100%)	
	p.V600E	1 (1%)	1 (2%)	0 (–)	0 (–)	0 (–)	
	Unknown	1 (1%)	1 (2%)	0 (–)	0 (–)	0 (–)	
*FGFR1*							0.619
	Wild	78 (84%)	38 (81%)	22 (92%)	9 (75%)	9 (90%)	
	Mutation	13 (14%)	8 (17%)	1 (4%)	3 (25%)	1 (10%)	
	Unknown	2 (2%)	1 (2%)	1 (4%)	0 (–)	0 (–)	
*EGFR*							0.692
	Wild	89 (96%)	45 (96%)	22 (92%)	12 (100%)	10 (100%)	
	Mutation	3 (3%)	1 (2%)	2 (8%)	0 (–)	0 (–)	
	Unknown	1 (1%)	1 (2%)	0 (–)	0 (–)	0 (–)	

*p < 0.05, statistically significant difference

Age: Kruskal-Wallis test, Others: Pearson's chi-square test

LGG, diffusely infiltrative glioma without histological features of anaplasia, which displays no/low mitotic activity; HGG, diffusely infiltrative glioma with histological features of anaplasia and displays significant mitotic activity; GBM features, microvascular proliferation or necrosis

### Clinical characteristics

There were 55 men (59%) and 38 women (41%) with a median age of 31 years (range 4–78 years). As shown in Figs. 2b and 3, only 26 patients were ≤ 18 years old (28%), and just seven patients were ≥ 70 years old (8%). According to the tumor locations, there seems to be significant difference in age distribution at other midline locations vs. the thalamus, the brainstem and the spinal cord locations (*p* = 0.041) (Table 2). As for sex, male predominance may exist in each location, but without significant difference (*p* = 0.809) (Table 2).

In MR images, gadolinium (Gd) enhancement of the tumor, as a high grade imaging feature, was observed in 68 tumors (73%) (Table 2 and Fig. 3). There was significant difference between groups (*p* = 0.016). Notably, Gd enhancement was not observed in 10 tumors (42%) in the brainstem group, a higher proportion than in the other groups. Hemorrhage was observed to have occurred in only one case in the thalamus [32].

Based on histopathological findings including morphology, cellularity, mitotic figures, and features of glioblastoma (GBM) (microvascular proliferation or necrosis) according to CNS WHO 2021 classification [57], 40 patients (43%) had GBM features and were diagnosed as having GBM. Thirty-six patients (39%) had diffusely infiltrative gliomas with histological features of anaplasia and displayed significant mitotic activity but without microvascular proliferation or necrosis, and they were diagnosed as having high-grade glioma (HGG) without GBM features. Sixteen patients (17%) had diffusely infiltrative glioma without histological features of anaplasia and displayed no/low mitotic activity without microvascular proliferation or necrosis, and they were diagnosed as having low-grade glioma (LGG). Approximately half of the cases with GBM features were in the thalamus and spinal cord groups (55% and 50%, respectively). Meanwhile, 79% of cases with LGG or HGG without features of GBM were in the brainstem group (Table 2).

Preoperative KPS scores ranged between 20 and 100 (median 70), and 46 patients had a score of ≥ 80 (49%). It may be notable that the preoperative KPS score was ≤ 70 in 67% cases in the spinal cord group. However, distribution of preoperative KPS score was not significantly different between tumor locations (*p* = 0.568).

Regarding EOR, 5 (5%), 11 (12%), 18 (19%), and 59 (63%) patients underwent GTR, STR, PR, and biopsy, respectively. Regardless of tumor locations, biopsy tended to be performed: it was performed in the thalamus, the brainstem, the spinal cord, and in other locations in 53%, 79%, 67% and 70% of cases, respectively (Table 1). EOR was not significantly different between tumor locations (*p* = 0.05). Surgical resection (46%) was more common in the thalamus group (46%) than in the other groups.

After surgery, 86 patients received adjuvant treatments of radiation (RT) and/or chemotherapy (93%). Although 82 patients underwent adjuvant RT, radiation was finally delivered for 86 patients (93% of the cohort), in which 82 patients (95%) and 74 patients (80%) received ≥ 40 Gy and ≥ 50 Gy, respectively (Table 2). The spinal cord group was significantly more likely to receive a lower radiation dose than other groups (*p* < 0.001). Chemotherapy was administered in 81 cases (87%), in which 76 patients received TMZ and only one patient in the thalamus group received ACNU with RT (Table 2, Fig. 3). BEV was administered with RT in 39 cases (42%). As shown in Table 2, adjuvant treatment regimen included RT + TMZ + BEV (35 cases, 38%), RT + TMZ (37 cases, 40%), RT + ACNU (1 case, 1%), RT + BEV (4 cases, 4%), RT alone (5 cases, 5%) and TMZ alone (4 cases, 4%), and were not significantly associated with tumor locations (*p* = 0.588). Meanwhile, bis-chloroethyl-nitrosourea wafers were placed in one case of the other midline location group. Tumor-treating fields therapy was applied in three cases in the thalamus in adult patients.

The observation period ranged between 0.5 and 63.5 months (median 15.6 months). During the observation period, tumor progression was observed in 58 patients (58/77, 75%). Repeat surgical resection was performed in seven cases (7/58, 12%). According to tumor locations, 6 of the 30 patients with a tumor in the thalamus and 1 of the 15 patients with a tumor in the brainstem underwent repeat resection [22] (Table 2, Fig. 3).

### Molecular characteristics

As shown in Table 2 and Fig. 3, *HIST1H3B* p.K27M mutation was observed in only two cases in the thalamus (2%) and all other cases had *H3F3A* p.K27M mutation (98%). *IDH1/2* was wild-type in all cases, regardless of the tumor location. *TERT* promoter mutations were observed in only three cases in the thalamus (3%). *MGMT* promoter methylation was found in nine cases (10%) across tumor locations: five cases in the thalamus (11%), one case in the brainstem (4%), one case in the spinal cord, and two cases in other locations (20%), but there was no statistical difference (*p* = 0.304). *TP53* mutation was detected in approximately half of cases across tumor locations (57%); there was no statistically significant difference (*p* = 0.207). *BRAF* p.V600E was observed in only one case in the thalamus (1%). This patient had co-occurrence of H3 p.K27M and *BRAF* p.V600E mutations. *FGFR1* mutation was found in 13 cases across tumor locations (14%), but there was no significant difference in frequency between the four locations (*p* = 0.619). Moreover, there was no significant difference between brainstem location (n = 24) and non-brainstem locations (n = 69) (*p* = 0.215). Notably, *FGFR1* mutations were observed in almost all adult cases with the exception of one pediatric case in the brainstem (Fig. 3). In the cases harboring *FGFR1* mutation, *TP53* mutation occurred in five cases (5/13, 38%). *EGFR* mutation was observed in three patients (3%) (one in the thalamus, two in the brainstem). These cases can be diagnosed as DMG, *EGFR*-mutant, one subtype in DMG, H3 K27-altered.

### Treatment outcomes and prognostic factors

**Fig. 4:**
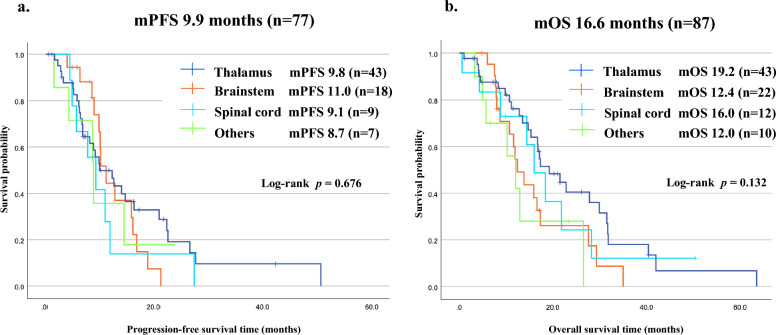
Kaplan–Meier survival curves according to tumor locations. **a** Median progression-free survival of the cohort (n = 77) was 9.9 ± 1.0 (7.9–11.9, 95% CI) months. Thalamus (n = 43), 9.8 months; Brainstem (n = 18), 11.0 months; Spinal cord (n = 9), 9.1 months; Others (n = 7), 8.7 months. **b** Median overall survival (mOS) of the cohort (n = 87) was 16.6 ± 1.4 (13.9–19.3, 95% CI) months. Thalamus (n = 43), 19.2 months; Brainstem (n = 22), 12.4 months; Spinal cord (n = 12), 16.0 months; Others (n = 10), 12.0 months

**Fig. 5:**
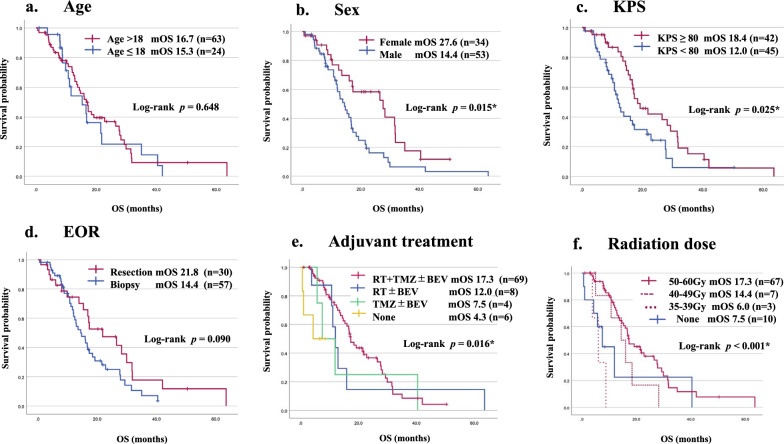
Kaplan–Meier survival curves according to clinical factors: age (**a**), sex (**b**), preoperative KPS score (**c**), extent of surgical resection (**d**) adjuvant treatment (**e**) and radiation dose (**f**) in the study cohort

PFS was reported in 77 cases, and OS was reported in 87 cases. Tumor progression was observed in 58 patients (58/77, 75%). Sixty patients had died by the time of analysis (60/87, 69%). Median PFS was 9.9 months, and median OS (mOS) was 16.6 months (Fig. 4). This was similar to previous reports regarding the mOS (Additional file 2: Table S2). There was no significant difference in PFS or OS between the four tumor location groups (*p* = 0.676 and 0.132, respectively, Fig. 4). Patients (> 18 years) did not have significantly different OS (16.7 months) compared with 15.3 months in those ≤ 18 years old (*p* = 0.648) (Fig. 5a, Table 3). Women had significantly longer OS than men (27.6 vs. 14.4 months) (*p* = 0.015) (Fig. 5b, Table 3). In analysis based on specific locations, any differences were without significance: the thalamus (*p* = 0.116), the brainstem (*p* = 0.115), the spinal cord (*p* = 0.234), other locations (*p* = 0.274) (Additional files 6, 7, 8, 9: Figures S3b, S4b, S5b, S6b). As for histopathological findings, there was no significant difference in OS between the LGG group (27.6 months), the HGG without features of GBM group (12.4 months), and the HGG with features of GBM group (17.1 months) (*p* = 0.546). Moreover, the group with GBM features did not have significantly different OS (17.1 months) compared with the LGG and HGG without features of GBM groups (14.4 months) (*p* = 0.069) (Additional file 5: Figure S2a, b, Table 3). Patients with preoperative KPS score of < 80 survived for a shorter time than those with KPS 80–100 (12.0 vs. 18.4 months) (*p* = 0.025) (Fig. 5c, Table 3), while those with preoperative KPS score of < 70 survived without significant difference to those with KPS score 70–100 (12.8 vs. 17.3 months) (*p* = 0.086) (Additional file 5: Figure S2c). Patients in the group that underwent surgical resection (GTR + STR + PR) tended to survive longer than those who received biopsy (21.8 vs. 14.4 months), but this difference was not significant (*p* = 0.090) (Fig. 5d, Table 3). There was no significant survival difference between GTR + STR and PR + biopsy groups (*p* = 0.060), but patients in the GTR + STR group tended to survive longer than those in the PR + biopsy group (29.9 vs. 14.7 months) (Additional file 5: Figure S2d, Table 3). The repeat surgical resection group tended to have prolonged OS compared with those without surgical resection (31.5 vs. 16.7 months) (*p* = 0.104) (Additional file 5: Figure S2e). Patients who received adjuvant RT + TMZ ± BEV had significantly longer OS than those who received RT ± BEV or TMZ ± BEV (17.3 vs. 12.0 or 7.5 months) (*p* = 0.016) (Fig. 5e). RT + TMZ ± BEV group had significantly longer OS than others (*p* = 0.031) (Additional file 5: Figure S2f, Table 3). As for TMZ therapy, there was no significant survival difference between the TMZ (+) and (−) groups (17.3 vs. 11.1 months) (*p* = 0.08) (Additional file 5: Figure S2g, Table 3), and regarding BEV therapy, there was no significant difference in survival between the BEV (+) and (−) groups (16.6 vs. 16.0 months) (*p* = 0.933) (Additional file 5: Figure S2h). This was also similar to the trend in the adjuvant phase (*p* = 0.477) (Additional file 5: Figure S2i). There was no significant difference in survival between the RT(+) and RT(−) groups (16.8 vs. 7.5 months) (*p* = 0.063), but the RT(+) group tended to survive longer than the RT(−) group (Additional file 5: Figure S2j, Table 3).

**Table 3:** Univariate and multivariate survival analysis of histone H3 K27-mutant diffuse midline glioma patients (n = 87)

Factors	Univariate analysis	Multivariate analysis	
	*p* value (log rank test)	HR (95%Cl)	*p* value
Age (> 18 vs. ≤ 18)	0.648		
Sex (Male vs. Female)	0.015*	2.22 (1.24–3.95)	0.007*
Histopathology (GBM features vs. Others)	0.069		
Preoperative KPS score (≥ 80 vs. < 80)	0.025*	0.45 (0.26–0.79)	0.006*
Extent of resection (≥ 80% vs. < 80%)	0.06		
Extent of resection (Resection vs. Biopsy)	0.09		
Radiation + Temozolomide (Yes vs. No)	0.031*	0.94 (0.37–2.42)	0.905
Radiation (Yes vs. No)	0.063		
Temozolomide (Yes vs. No)	0.08		
Radiation dose (≥ 50Gy vs. < 50Gy)	0.008*	0.45 (0.19–1.10)	0.079
*TERT* promoter (Wild vs. Mutant)	0.533		
*MGMT* promoter (Met vs. Unmet)	0.967		
*TP53* (Wild vs. Mutant)	0.754		
*BRAF* (Wild vs. p.V600E)	–		
*FGFR1* (Wild vs. Mutant)	0.311		
*EGFR* (Wild vs. Mutant)	0.638		

*p < 0.05, statistically significant difference

Regarding the RT dose, there was significant difference in mOS between the groups (*p* < 0.001) (Fig. 5f). Median OS of RT ≥ 50 Gy group was the longest among the groups (17.3 months), and the difference with < 50 Gy groups (10.7 months) reached statistical significance (*p* = 0.008) (Additional file 5: Figure S2k, Table 3). Notably, there was also a statistical difference between ≥ 40 Gy and < 40 Gy groups (17.1 vs. 7.5 months) (*p* = 0.006) (Additional file 5: Figure S2l). With the exception of the spinal cord group, there was significant difference in mOS between the groups (*p* < 0.001) (Additional file 10: Figure S7a). Median OS of RT ≥ 50 Gy group was the longest between the groups (17.1 months), and the difference with < 50 Gy groups (7.5 months) reached statistical significance (*p* = 0.031) (Additional file 10: Figure S7b). However, there was no statistical difference between ≥ 40 Gy and < 40 Gy groups (16.8 vs. 7.5 months) (*p* = 0.144) (Additional file 10: Figure S7c).

**Fig. 6:**
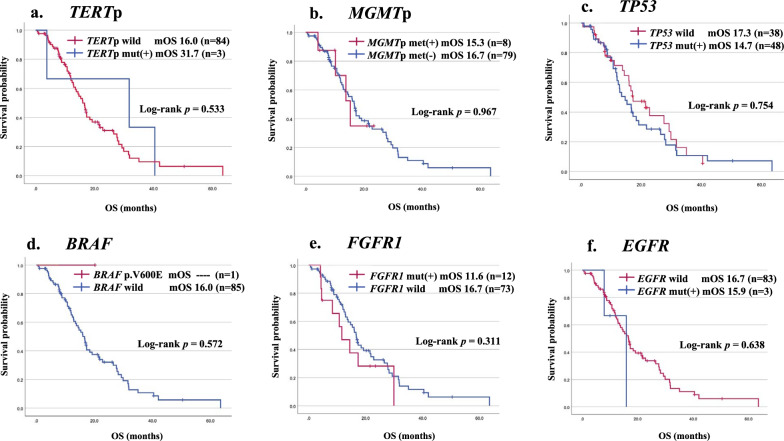
Kaplan–Meier survival curves according to molecular factors: *TERT* (**a**), *MGMT* (**b**), *TP53* (**c**), *BRAF* (**d**), *FGFR1* (**e**) and *EGFR* (**f**) in the study cohort

Regarding molecular status, *TERT* promoter mutation status showed no significant difference in OS between wild-type (16.0 months) and mutated (31.7 months) groups (*p* = 0.533). However, the mutated group had too small a population (n = 3) to compare with the wild-type group (n = 84) (Fig. 6a, Table 3). Similarly, *MGMT* promoter methylated group (n = 8) did not have significant difference in OS compared with the unmethylated group (n = 79) (15.3 vs.16.7 months) (*p* = 0.967) (Fig. 6b, Table 3). As for *TP53* status, no significant difference was found in OS between wild-type and mutated groups (17.3 vs. 14.7 months) (*p* = 0.754) (Fig. 6c, Table 3). The *BRAF* V600E group (n = 1) was too small for statistical analysis (Fig. 6d, Table 3). *FGFR1* mutated group (n = 12) did not show longer OS than the wild-type group (n = 73) (11.6 vs.16.7 months) (*p* = 0.311) (Fig. 6e, Table 3). In the *EGFR* mutated group, a number of cases (n = 3), were shown to have shorter OS than the wild-type group (15.9 vs. 16.7 months) (*p* = 0.638) (Fig. 6f, Table 3).

We conducted a subgroup analysis of clinical and genetic prognostic factors for each location (Additional files 6, 7, 8, 9: Figures S3, S4, S5, S6). When stratifying by sex, no significant difference was observed in any of the locations (Additional files 6, 7, 8, 9: Figures S3b, S4b, S5b, S6b). In the thalamus, there was a significant difference in patients with KPS ≥ 80 and radiation dose (Additional file 6: Figure S3c, f). Similarly, in the brainstem, significant difference was observed in radiation dose (Additional file 7: Figure S4f). For the spinal cord and other midline locations, the sample size was small, potentially compromising the reliability of the observed significance (Additional files 8, 9: Figures S5, S6).

As the results of univariate analysis of the relationships between characteristics and estimated survival times for all cases of DMG, female sex, preoperative KPS score of ≥ 80, adjuvant RT + TMZ treatment and RT dose (≥ 50 Gy) were significantly associated with longer OS (Table 3).

The results of multivariate analysis of factors associated with OS are also shown in Table 3. Independent factors for good prognosis in the present cohort were identified as female sex and preoperative KPS score of ≥ 80.

## Discussion

For the present study, we reviewed histone H3 K27M-mutant diffuse gliomas located at the midline structures in the Kansai Network dataset. We found 93 patients with midline DMG (47 in the thalamus, 24 in the brainstem, 12 in the spinal cord, and 10 in other midline locations). A separate article will report on non-midline tumors in more detail. The results of this study could be said to be representative of the current state of clinical practice for patients with DMG and molecular analyses of DMG in real-world settings.

### Tumor location

Diffuse midline glioma, H3 K27-altered is defined as a tumor found in the thalamus, brainstem, spinal cord, and occasionally in the pineal gland, the hypothalamus or the cerebellum [57]. However, in clinical practice, H3 K27M-mutant diffuse gliomas could exist at the anatomically non-midline location. As shown in Additional file 1: Table S1, the definition of midline may have been confused in previous studies of DMG. For example, a tumor located at corpus callosum or basal ganglia was considered to be a midline tumor by some researchers, but as a non-midline tumor by others [1, 2, 7, 11, 19, 23, 25, 31, 39, 40, 42, 51, 55, 60, 62]. Diffuse glioma located at the thalamus along with the basal ganglia or both the thalamus and the corpus callosum was included in the studies of DMG [25, 28, 56]. The basal ganglia, embryologically associated with the cerebral cortex, is sometimes the location in which diffuse hemispheric glioma, H3 G34-mutant arise [19, 60]. From developmental and anatomical points of view, the cerebrum including the corpus callosum and the basal ganglia may be usually considered as non-midline structures [45, 49, 50]. However, a thalamic glioma involving the basal ganglia or corpus callosum would be categorized within the DMG [25, 28, 56]. On the other hand, there are some reports of the cerebral cortex being included in the location of the DMG [31, 38, 42, 51, 55, 60]. As for cerebellum, the vermis is apparently located at the midline, but diffuse glioma at the cerebellar hemisphere have sometimes been classified as non-midline tumors [21]. Meanwhile, a tumor in the ventricle was included in several DMG studies, although the ventricle was not described in CNS WHO 2021 [1, 2, 7, 14, 20, 31, 33, 47, 55, 57, 62]. Additionally, one study of DMG included diffuse glioma in the suprasellar region [51, 62]. For diffuse glioma extending from the spinal cord to the thalamus, one report introduced the concept of ‘diffuse growth along with brain axis’ [14]. Others have used the term ‘whole-brain type lesions’ for widespread lesions involving three or more contiguous lobes in the brain, and involvement of one or more traditional midline structures [39].

Based on these previous reports, we classified tumors in which the primary location was identified in the ventricles as ‘other midline locations’ (Table 1). Furthermore, among tumors which primarily involved the corpus callosum or basal ganglia, those which predominantly involved midline structures were classified as other midline locations, and tumors that primarily included the cerebral hemisphere were classified as non-midline, respectively (Table 1). In cases of non-contiguous, multifocal lesions where the primary location was indeterminate, we classified them as other midline locations if the main area involved midline structures, and as non-midline if it involved the cerebral hemispheres (Table 1). Using these criteria, we excluded 16 non-midline cases of 109 patients with H3 K27M-mutant diffuse glioma in Kansai Network cohort, as described in the Material and Methods section above. However, tumor locations of DMGs are sometimes heterogenous and complicated, so it may be difficult to identify the true tumor origin. We therefore suggest one standard definition for DMGs. However, this may still be incomplete, and future validation and reconsideration will be needed using a larger cohort, which we believe will improve the understanding of the features of DMGs.

### Age

DMG is categorized in the pediatric-type diffuse high-grade gliomas of CNS WHO 2021; however, DMG may occur in adults, as well as in children and adolescents [14, 24, 26, 27, 33, 35, 42–44, 51, 55, 56, 60–62]. Previously, not-so-small percentages of adult cases were included in studies of DMG. For example, a recent study by Zheng et al. contained 57.3% patients aged ≥ 19 years, and other research by Williams et al. enrolled 48.6% patients aged ≥ 20 years [58, 62]. In our study cohort, the percentage of patients aged ≥ 19 years was 72.0%, so it may be higher than that of previous studies. There may be a higher occurrence in adults compared with in children [14, 56]. However, it should be taken into account that the limited number of pediatric cases may be due to the lower amount of surgical tissue sampling for brainstem tumors, which are more common in children than in adults [57]. DMG is generally thought to occur more commonly in children, but given the larger adult population, it is believed that the number of adult cases has become more prevalent as a result. DMG should nonetheless be considered as the differential diagnosis of adult diffuse gliomas.

### Sex

Gliomas are known to have higher incidence and poorer prognosis in men [34, 48]. Numerous studies have indicated that women have a better prognosis than men, with factors such as hormones, metabolism, the immune system, genetic and molecular mechanisms, neurogenic niches and therapeutic responsiveness, among other factors, being suggested as reasons for this [8, 48]. None of the previous DMG reports found a significant difference in the prognosis by sex [23, 56, 62]. This study is thus the first report to list female sex a favorable prognostic factor in DMG.

### Histopathological characteristics

In this study, histopathological features of DMGs were varied, and diagnosed as LGG (17%), HGG without features of GBM (39%) or HGG with features of GBM (43%). Zheng et al. reported common observations of microvascular proliferation (77/164, 47.0%), tumor cell necrosis (53/164, 32.3%), and multinucleated tumor cells (38/164, 23.2%) [62]. In this study, we observed similar microvascular proliferation (21/92, 23%), and tumor cell necrosis (28/92, 30%). These findings indicate that DMGs may show predominantly HGG or GBM histopathological features. On the other hand, some tumors showed LGG characteristics in morphology, despite their poor clinical prognosis. There might be a diagnostic limitation due to tiny biopsy specimens for DMG. Moreover, biopsies of low grade regions from tumors with high grade imaging features could be a potential confounder, especially in the biopsy cases; indeed, there were 24 cases (45%) in this study, comprising 11 cases in the thalamus group (50%), six cases in the brainstem group (31%), four cases in the spinal cord group (67%) and three cases in the others group (50%) (Additional file 3: Table S3). However, the present findings may indicate that H3 K27M-mutation does not always induce malignant histopathological phenotypes. Significance of histological malignant transformation occurring in DMGs therefore requires examination in future studies in combination with molecular analysis.

### Molecular features

Regarding diagnostic molecular pathology, CNS WHO 2021 Blue Book stated that co-occurrence of histone H3 K27 mutation with *IDH* mutations is exceptional; correspondingly, all cases revealed *IDH* wildtype in our genetic analysis [57]. Similarly, *TERT* promoter mutations and *MGMT* promoter methylation represent rare events in DMGs. However, *TERT* mutated and *MGMT* methylated were detected in 3% and 9% of our cases, respectively, and these were mainly in the thalamus [57].

Only one patient in our cohort (a 4-year-old girl) had bilateral thalamic tumors harboring *HIST1H3B* p.K27M and *EGFR* mutations (Additional file 4: Figure S1B)*.* As described in the WHO Blue Book, bi-thalamic tumors are more common in the *EGFR*-subtype of DMGs, most often occurring during childhood, with median age of 7–8 years [57].

Histone H3 K27M mutations are generally found to be associated with collaborating mutations of canonical cancer-associated pathways [57]. For example, *TP53* mutations were found in 57% of our study cohort, being detected predominantly in H3.3 p.K28M (K27M)-mutant and *EGFR*-mutant cases according to a previous report [57]. *BRAF* p.V600E mutation co-occurred in just one case (1%) in this study with H3.3 p.K28M (K27M) mutation [57]. Gain-of-function mutation and genetic amplification of growth factor receptor involved in brain development are said to be common in H3 K27M-mutant DMGs, and *FGFR1* mutation was found in 14% of patients in the present study [57]. A recent comprehensive genomic study of *H3F3A*-mutant high-grade gliomas revealed that *FGFR1* hotspot point mutations (N546K and K656E) were exclusively identified in H3 K27M-mutant DMGs (64/304, 21%); these tend to occur in older patients (median age: 32.5 years) and mainly arise in the diencephalon [54, 58]. In this study, *FGFR1* mutations were mainly observed outside of the brainstem, replicating the findings reported by Williams et al. [58]. The above findings were also similar to those observed in Japanese cases, and demonstrating a similar trend. Mutations were reportedly suggested to be associated with a favorable prognosis, and *FGFR1* mutations are mutually exclusive with *TP53* mutation [43]. *TP53* mutations are associated with a poor prognosis [5, 43, 56]. However, these trends were not observed in our study cohort; these differences in prognostic factors and variations may be attributed to racial disparities. A future study will aim to validate these points within a larger sample size.

### Relevance to treatments

Standard of care for DMG has never been determined, but several treatment options have been suggested, regardless of evidence. Surgical resection of DMG is often difficult, and in our cohort, biopsy tended to be undertaken (63%). However, aggressive resection may be attempted if feasible, and there were few cases in our cohort in which GTR was actually possible (5%) [23]. On the other hand, adjuvant RT + TMZ was conducted in the majority of our cohort (78%). Radiotherapy has been regarded as an important treatment option for brainstem gliomas, as is DMG [23]. TMZ concomitant with and adjuvant to RT is a widely used approach to GBM, but the role in cases of DMG has never been demonstrated [12, 46]. In our series, BEV was administered in 57% of cases, and there is a previous report of effectivity [59].

RT has been suggested in several studies to prolong the patients’ survival, although there is also a report to the contrary that radiotherapy does not influence prognosis [6, 23, 56]. Regarding the radiation dose, a standard protocol for DMG has never been established, but it often ranges from 36 to 65 Gy [6, 36, 42]. In our study cohort, 80% of patients received 50–60 Gy. The spinal cord group, however, was likely to receive a lower radiation dose (< 50 Gy), probably due to a spinal cord tolerance dose of < 50 Gy, and to avoid potential adverse effects such as bone marrow suppression in the long lesions. As for the prognostic impact of RT, survival benefit was demonstrated when we used ≥ 50 Gy for patients of our study.

### Prognostic factors

The treatment outcomes of our series are mostly consistent with those of previous reports (Additional file 2: Table S2). To date, several prognostic factors of DMGs have been suggested (Additional file 2: Table S2). Clinical factors such as age, sex, tumor location, tumor size, EOR and radiation have been considered in some reports [6, 13–16, 23–25, 28, 37, 42–44, 53, 56, 62]. As for pathological and molecular factors, there has been previous discussion of histological grading, Ki-67 labelling index, histone H3 subtype and mutations of *EZH2,TP53*, *ATRX*, *TERT* promoter, *BRAF* and *FGFR1* [3, 6, 9, 13–16, 23–25, 28, 37, 42–44, 53, 56, 62]. As for tumor locations, brainstem location is reportedly a poor prognostic factor [16, 62], but in this cohort, there was no significant difference in OS between the four tumor location groups. Adulthood has also been reported as a good prognostic factor [43, 44, 54], but in this cohort, there was no significant difference in OS between adults and infants. As for sex, it was not previously reported to be a prognostic factor, but we found female sex to be an independent factor in good prognosis. Meanwhile, for pathological findings, no significant difference was found among WHO grade 2,3,4 for prognosis [62], and we obtained similar results in this study as well. As for molecular factors, *EZH2* expression, *TP53* mutation, *ATRX* expression, are reportedly poor prognostic factors and *FGFR1* mutation is reportedly a good prognostic factor [24, 43, 56], but we found no significant difference in OS between *TP53* mutations in our cohort. We did not investigate *EZH2* and *ATRX* expression in this cohort. RT is reportedly a good prognostic factor [44, 56], and similarly we found RT ≥ 50 Gy to be a good prognostic factor in this cohort.

As for the prognostic impact of each factor, however, consistent results cannot be achieved universally through studies; the limited number of study patients could partly explain the absence of statistical power to detect differences between groups. In our series, there was no statistically significant difference in OS according to age, location, resection, histological grading or genetic status (Table 3). On the other hand, our multivariate analysis identified female sex and preoperative KPS score ≥ 80 as independent prognostic factors (Table 3). Further investigation in a larger cohort could contribute to a better understanding of the prognostication of DMGs.

### Summary of the present study and future challenges

Complete resection of DMGs without inducing new neurological deficits is challenging. In this study, no significant difference in OS was observed based on the resection rate, but a significant difference in OS was found based on the radiation dose. It is considered crucial to complete radiation therapy without compromising KPS through surgery as a treatment. We identified no significant prolonging of OS in cases with *FGFR1* mutations, but the development of local treatment with molecular targeted drugs is desired.

## Limitations

Owing to the multi-institutional retrospective cohort design, this study has several limitations. Unlike in a randomized study, there could be selection bias regarding the distribution of tumor locations and decision-making of treatment strategy. The limited number of patients could explain the lack of statistical power to detect differences between groups. Attending physicians may decide to deliver treatments with consideration of the patients’ age, conditions and wishes, and thus patient selection could affect the survival findings. Variation of treatment regimen at multiple institutions, such as radiation protocol and dose schedule, should also be considered. The modest prognostic impact of clinical and molecular characteristics might be partly due to the limited population.

Our Kansai classification has limitations. The ambiguity of the current midline terminology in DMG allowed us to discriminate between the midline and non-midline structures for definition of DMG. However, it is challenging to determine the location of the origin of DMG. Some tumors which appear centered in the hemispheres have involvement of midline structures. There is the possibility, for example, of a tumor starting in the midline, but from which cells that migrated outward ultimately formed the most aggressive-appearing regions according to images. Any diffuse glioma with H3 K27M mutation would qualify for the diagnosis of DMG. Further studies could help to clarify this problem. It would nonetheless be better to consider that the Kansai classification is our approach in this study for better understanding of the pathology of DMG.

There are also limitations in this study regarding the discrimination between the midline and non-midline structures for definition of DMG. In our Kansai classification, the basal ganglia and corpus callosum were categorized as non-midline structures and were excluded from the analysis of this study, although tumors located at the basal ganglia or corpus callosum but mainly involving midline structures such as the thalamus or brainstem were categorized as midline tumors (Table 1). In the notion that any diffuse glioma with H3 K27M mutation would qualify for the diagnosis of DMG, the current midline terminology in DMG would not be necessary. Further studies of diffuse non-midline gliomas including the basal ganglia and corpus callosum tumors could help to clarify this problem.

## Conclusions

Considering the term “midline” areas, which had been confused in previous reports, we classified four midline locations based on previous reports and anatomical findings in this study, and reported characteristics and outcomes of patients with histone H3 K27M-mutant DMG in the Kansai Network. This community-based study is suggested to be representative of the present status of real-world practice. Further investigation in a larger patient population could contribute to better understanding of the pathology of DMG.

## Supplementary Information

**Additional file 1: Table S1.** Classification of "midline" or "non-midline" in the previous reports.

**Additional file 2: Table S2.** Summary of the previous reports on histone H3 K27-mutant diffuse midline glioma cohort studies.

**Additional file 3: Table S3.** Discordance between imaging features (low/high grade) and histological findings (presence or absence of GBM features) in the biopsy cases.

**Additional file 4: Figure S1**. MR imaging of histone H3 K27-mutant gliomas in Kansai Network. A. a, b: FLAIR, The tumor is a sole lesion, and main location is the third ventricle: Others/midline (included). B. a, b, c: FLAIR, The tumor is comprised of contiguous multifocal lesions, and main location is the thalamus: Thalamus/midline (included). C. a: FLAIR, b:T1-Gd, The tumor is comprised of non-contiguous multifocal lesions, and the main location is the thalamus and/or corpus callosum, unclassified tumor: Others/midline (included). D. a: T1-Gd, b, c: FLAIR, The tumor is comprised of non-contiguous multifocal lesions, and the main location is unclassified, the cerebral hemisphere is more involved than the brainstem : Others/non-midline (excluded). E. a, b: FLAIR, c:T1-Gd, The tumor is comprised of contiguous multifocal lesions, and the main location is the left basal ganglia, which involve the thalamus and/or the brainstem more than the cerebral hemisphere: Others/midline (included). F. a: FLAIR, b:T1-Gd, The tumor is comprised of contiguous multifocal lesions, and the main location is the right medial temporal lobe: Cerebral hemisphere/non-midline (excluded)

**Additional file 5: Figure S2**. Kaplan–Meier survival curves according to clinical factors: histopathology (LGG vs. HGG without GBM features vs. GBM features) (a), histopathology (LGG + HGG without GBM features vs. GBM features) (b), preoperative KPS score (≥ 70 vs. < 70) (c), EOR (GTR + STR vs. PR + Biopsy) (d), repeat surgical resection (e), RT+TMZ (f), TMZ (g), BEV (adjuvant + recurrent) (h), BEV (adjuvant) (i), Radiation (j), RT (≥ 50 Gy vs. < 50 Gy) (k), RT (≥ 40 Gy vs. < 40 Gy) (l).

**Additional file 6: Figure S3**. (Thalamus). Kaplan–Meier survival curves according to clinical factors: age (a), sex (b), preoperative KPS score (c), extent of surgical resection (d) adjuvant treatment (e) and radiation dose (f), molecular factors: TERT (g), MGMT (h), TP53 (i), BRAF (j), FGFR1 (k) and EGFR (l) in the study cohort.

**Additional file 7: Figure S4**. (Brainstem). Kaplan–Meier survival curves according to clinical factors: age (a), sex (b), preoperative KPS score (c), extent of surgical resection (d) adjuvant treatment (e) and radiation dose (f), molecular factors: TERT (g), MGMT (h), TP53 (i), BRAF (j), FGFR1 (k) and EGFR (l) in the study cohort.

**Additional file 8: Figure S5**. (Spinal cord). Kaplan–Meier survival curves according to clinical factors: age (a), sex (b), preoperative KPS score (c), extent of surgical resection (d) adjuvant treatment (e) and radiation dose (f), molecular factors: TERT (g), MGMT (h), TP53 (i), BRAF (j), FGFR1 (k) and EGFR (l) in the study cohort.

**Additional file 9: Figure S6**. (Other midline location). Kaplan–Meier survival curves according to clinical factors: age (a), sex (b), preoperative KPS score (c), extent of surgical resection (d) adjuvant treatment (e) and radiation dose (f), molecular factors: TERT (g), MGMT (h), TP53 (i), BRAF (j), FGFR1 (k) and EGFR (l) in the study cohort.

**Additional file 10: Figure S7**. Kaplan–Meier survival curves according to radiation dose without spinal cord group: Radiation dose (a), RT (≥ 50 Gy vs. < 50 Gy) (b), RT (≥ 40 Gy vs. < 40 Gy) (c)

## Data Availability

The datasets analyzed in the current study are available from the corresponding authors upon reasonable request.

## Abbreviations

ACNU: Nimustine hydrochloride
ATRX: Alpha-thalassemia/mental retardation, X-linked
BEV: Bevacizumab
BRAF: V-raf murine sarcoma viral oncogene homolog B1
CI: Confidence interval
CNS: Central Nervous System
CNS WHO 2021: The 5th edition of the World Health Organization classification of Tumors of the Central Nervous System
DMG: Diffuse midline glioma
EGFR: Epidermal growth factor receptor
EOR: Extent of surgical resection
EZH2: Enhancer of zeste homolog2
FLAIR: Fluid Attenuated Inversion Recovery
FGFR1: Fibroblast Growth Factor Receptor 1
GBM: Glioblastoma
Gd: Gadolinium
GTR: Gross total resection
Gy: Gray
HGG: High grade glioma
HR: Hazard ratio
IDH: Isocitrate dehydrogenase
Kansai Network: Kansai Molecular Diagnosis Network for CNS Tumors
KPS: Karnofsky performance status
LGG: Low grade glioma
Met: Methylated
MGMT: O-6-Methylguanine-DNA Methyltransferase
mOS: Median overall survival
Mut: Mutated
OS: Overall survival
PR: Partial resection
PFS: Progression free survival
RT: Radiation, Radiotherapy
STR: Subtotal resection
TERT: Telomerase reverse transcriptase
TMZ: Temozolomide
TP53: Tumor protein p53
