# Correction: Neuroradiological, genetic and clinical characteristics of histone H3 K27-mutant diffuse midline gliomas in the Kansai Molecular Diagnosis Network for CNS Tumors (Kansai Network): multicenter retrospective cohort

**DOI:** 10.1186/s40478-024-01863-3

**Published:** 2024-10-01

**Authors:** Nobuhide Hayashi, Junya Fukai, Hirokazu Nakatogawa, Hiroshi Kawaji, Ema Yoshioka, Yoshinori Kodama, Kosuke Nakajo, Takehiro Uda, Kentaro Naito, Noriyuki Kijima, Yoshiko Okita, Naoki Kagawa, Yoshinobu Takahashi, Naoya Hashimoto, Hideyuki Arita, Koji Takano, Daisuke Sakamoto, Tomoko Iida, Yoshiki Arakawa, Takeshi Kawauchi, Yukihiko Sonoda, Yuta Mitobe, Kenichi Ishibashi, Masahide Matsuda, Takamune Achiha, Takahiro Tomita, Masahiro Nonaka, Keijiro Hara, Noriyoshi Takebe, Takashi Tsuzuki, Yoshikazu Nakajima, Shiro Ohue, Nobuyuki Nakajima, Akira Watanabe, Akihiro Inoue, Masao Umegaki, Daisuke Kanematsu, Asako Katsuma, Miho Sumida, Tomoko Shofuda, Masayuki Mano, Manabu Kinoshita, Kanji Mori, Naoyuki Nakao, Yonehiro Kanemura

**Affiliations:** 1 Department of Neurosurgery, Wakayama Rosai Hospital, Kinomoto 93-1, Wakayama City, Wakayama 640-8505 Japan; 2 Kansai Molecular Diagnosis Network for CNS Tumors, Osaka City, Osaka 540-0006 Japan; 3 Department of Neurological Surgery, School of Medicine, Wakayama Medical University, Kimiidera 811-1, Wakayama City, Wakayama 641-8510 Japan; 4 Department of Pediatric Neurosurgery, Seirei Hamamatsu General Hospital, Hamamatsu, Shizuoka 430-8558 Japan; 5 Department of Neurosurgery, Seirei Hamamatsu General Hospital, Hamamatsu, Shizuoka 430-8558 Japan; 6 Division of Molecular Medicine, Department of Biomedical Research and Innovation, Institute for Clinical Research, NHO Osaka National Hospital, Osaka City, Osaka 540-0006 Japan; 7 Department of Diagnostic Pathology and Cytology, Osaka International Cancer Institute, Osaka City, Osaka 541-8567 Japan; 8 Department of Neurosurgery, Osaka Metropolitan University Graduate School of Medicine, Osaka City, Osaka 545-8585 Japan; 9 Department of Neurosurgery, Osaka University Graduate School of Medicine, Suita, Osaka 565-0871 Japan; 10 Department of Neurosurgery, School of Medical Science, Kyoto Prefectural University Graduate, Kyoto City, Kyoto 602-8566 Japan; 11 Department of Neurosurgery, Osaka International Cancer Institute, Osaka City, Osaka 541-8567 Japan; 12 Department of Neurosurgery, Hyogo College of Medicine, Nishinomiya, Hyogo 663-8501 Japan; 13 Department of Neurosurgery, Kyoto University Graduate School of Medicine, Kyoto City, Kyoto 606-8507 Japan; 14 Department of Neurosurgery, Osaka Red Cross Hospital, Osaka City, Osaka 543-8555 Japan; 15 Department of Neurosurgery, Faculty of Medicine, Yamagata University, Yamagata City, Yamagata 990-8560 Japan; 16 Department of Neurosurgery, Osaka City General Hospital, Osaka City, Osaka, 534-0021 Japan; 17 Department of Neurosurgery, Faculty of Medicine, University of Tsukuba, Tsukuba, Ibaraki 305-8575 Japan; 18 Department of Neurosurgery, Kansai Rosai Hospital, Amagasaki, Hyogo 660-8511 Japan; 19 Department of Neurosurgery, Graduate School of Medicine and Pharmaceutical Sciences, University of Toyama, Toyama City, Toyama, 930-0194 Japan; 20 Department of Neurosurgery, Kansai Medical University, Hirakata, Osaka 573-1191 Japan; 21 Department of Neurosurgery, Tokushima University Graduate School of Biomedical Sciences, Tokushima City, Tokushima, 770-8501 Japan; 22 Department of Neurosurgery, Medical Research Institute, Tazuke Kofukai Foundation, Kitano Hospital, Osaka City, Osaka, 530-8480 Japan; 23 Department of Neurosurgery, Sakai City Medical Center, Sakai, Osaka 593-8304 Japan; 24 Department of Neurosurgery, Kobe Tokushukai Hospital, Kobe, Hyogo 655-0017 Japan; 25 Department of Neurosurgery, Ehime Prefectural Central Hospital, Matsuyama, Ehime 790-0024 Japan; 26 Department of Neurosurgery, Tokyo Medical University, Tokyo, 160-0023 Japan; 27 Department of Neurosurgery, Kindai University Nara Hospital, Ikoma, Nara 630-0293 Japan; 28 Department of Neurosurgery, Ehime University School of Medicine, Toon, Ehime 791-0295 Japan; 29 Department of Neurosurgery, Suita Municipal Hospital, Suita, Osaka 564-8567 Japan; 30 Division of Regenerative Medicine, Department of Biomedical Research and Innovation, Institute for Clinical Research, NHO Osaka National Hospital, Osaka City, Osaka, 540-0006 Japan; 31 Division of Stem Cell Research, Department of Biomedical Research and Innovation, Institute for Clinical Research, NHO Osaka National Hospital, Osaka City, Osaka, 540-0006 Japan; 32 Department of Central Laboratory and Surgical Pathology, NHO Osaka National Hospital, Osaka City, Osaka, 540-0006 Japan; 33 Department of Neurosurgery, Asahikawa Medical University, Asahikawa, Hokkaido 078-8510 Japan; 34 Department of Neurosurgery, Yao Municipal Hospital, Yao, Osaka 581-0069 Japan; 35 Department of Neurosurgery, NHO Osaka National Hospital, Osaka City, Osaka, 540-0006 Japan

Acta Neuropathologica Communications (2024) 12:120.

10.1186/s40478-024-01808-w.

Following publication of the original article [1], the wrong Supplementary figures appeared in the published article; the Supplementary figure should have appeared as shown below:

Additional file 4, Additional file 5, Additional file 6, and Additional file 7 are swapped. The files should be reorganized and numbered as Supplementary Figure 1, Supplementary Figure 2, Supplementary Figure 3 and Supplementary Figure 4 respectively.

The original article has been corrected.
