# Supplementary material for: Neuroradiological, genetic and clinical characteristics of histone H3 K27-mutant diffuse midline gliomas in the Kansai Molecular Diagnosis Network for CNS Tumors (Kansai Network): multicenter retrospective cohort
Source: Acta Neuropathol Commun. 2024 Jul 27;12:120. doi: 10.1186/s40478-024-01808-w (PMC11282756; doi:10.1186/s40478-024-01808-w)
Supplement: Supplementary file 1 — Additional file 1: Table S1. Classification of "midline" or "non-midline" in the previous reports. [file 40478_2024_1808_MOESM1_ESM.pdf]

# Supplementary Table 1

Table S1 Classification of "midline" or "non-midline" in the previous reports

| Location | "Midline" | "Non-Midline" |
|---|---|---|
| **Cerebral hemisphere** | Yoshimoto K et al. 2017, Brain Tumor Pathol. 103 [60]<br>Pratt D et al. 2018, Acta Neuropathol. 299 [38]<br>Wang L et al. 2018, Human Pathol. 89 [55]<br>Schreck KC et al. 2019, Journal of Neuro Oncol. 87 [42]<br>Maeda S et.al. 2020, Acta Neuropathol commun. 8 [31]<br>Thust S et al. 2021, Quant Imaging Med Surg. 43 [51] | Lopez G et al. 2017, Acta Neuropathol Commun. 38 [29]<br>Huang T et al. 2018, Oncotarget. 37112 [21]<br>Qiu T et al. 2020, J Neurosurg. 1662 [39]<br>Chia N et al. 2021 Neuro Oncol Adv. 1 [11] |
| **Cerebellum** | Aboian M.S. et al. 2017, AJNR Am J Neuroradiol. 795 [1]<br>Funata N et al. 2018, Brain Tumor Pathol. 29 [17]<br>Maeda S et.al 2020, Acta Neuropathol commun. 8 [31] | Huang T et al. 2018, Oncotarget. 37112 [21] |
| **Corpus callosum** | Aboian M.S. et al. 2017, AJNR Am J Neuroradiol. 795 [1]<br>Wang L et al. 2018, Human Pathol. 89 [55]<br>Schreck KC et al. 2019, Journal of Neuro Oncol. 87 [42]<br>Aboian M.S. et al. 2019, AJNR Am J Neuroradiol. 1804 [2]<br>Qiu T et al. 2020, J Neurosurg. 1662 [39]<br>Maeda S et.al 2020, Acta Neuropathol commun. 8 [31] | Rocca GL et al. 2019 World Neurosurg. 174 [40]<br>Chia N et al. 2021 Neuro Oncol Adv. 1 [11] |
| **Ventricle** | Solomon DA et al. 2016, Brain Pathol. 569 [47]<br>Aboian M.S. et al. 2017, AJNR Am J Neuroradiol. 795 [1]<br>Meyronet D et al. 2017, Neuro Oncol. 1127 [33]<br>Wang L et al. 2018, Human Pathol. 89 [55]<br>Bozkurt SU et al. 2018, Childs Nerv Syst. 107 [7]<br>Ebrahimi A et al. 2019, J Cancer Res Clin Oncol. 839 [14]<br>Aboian M.S. et al. 2019, AJNR Am J Neuroradiol. 1804 [2]<br>Maeda S et.al 2020, Acta Neuropathol commun. 8 [31]<br>Hassan U et al. 2021, Cureus. e17267 [20]<br>Zheng L et al. 2022, Am J Surg Pathol. 863 [62] | |
| Basal ganglia | Karremann M et al. 2018, Neuro Oncol. 123 [25]<br>Bozkurt SU et al. 2018, Childs Nerv Syst. 107 [7]<br>Maeda S et.al 2020, Acta Neuropathol commun. 8 [31]<br>Thust S et al. 2021, Quant Imaging Med Surg. 43 [51]<br>Zheng L et al. 2022, Am J Surg Pathol. 863 [62]<br>Jang SW et al. 2022, Brain Tumor Res Treat. 255 [23] | Gutierrez DR et al. 2020 Clin Cancer Res. 1856 [19]<br><br>(Yoshimoto K et al. 2017, Brain Tumor Pathol. 103)* [60] |
| **Supra-sella** | Thust S et al. 2021, Quant Imaging Med Surg. 43 [51]<br>Zheng L et al. 2022, Am J Surg Pathol. 863 [62] | |
| **Diffuse growth along with brain axis †** | Ebrahimi A et al. 2019, J Cancer Res Clin Oncol. 839 [14] | |
| **Whole-brain type lesion ‡** | Qiu T et al. 2020, J Neurosurg. 1662 [39] | |

* G34R-positive at the basal ganglia, so "non-midline" classified

† Widespread lesions involving the spinal cord, brainstem and thalamus

‡ Widespread lesions involving the three or more contiguous lobes in the brain, and involvement of one or more traditional midline structures
