# Supplementary material for: Neuroradiological, genetic and clinical characteristics of histone H3 K27-mutant diffuse midline gliomas in the Kansai Molecular Diagnosis Network for CNS Tumors (Kansai Network): multicenter retrospective cohort
Source: Acta Neuropathol Commun. 2024 Jul 27;12:120. doi: 10.1186/s40478-024-01808-w (PMC11282756; doi:10.1186/s40478-024-01808-w)
Supplement: Supplementary file 2 — Additional file 2: Table S2. Summary of the previous reports on histone H3 K27-mutant diffuse midline glioma cohort studies. [file 40478_2024_1808_MOESM2_ESM.pdf]

Supplementary Table 2

Table S2 Summary of the previous reports on histone H3 K27-mutant diffuse midline glioma cohort studies

Author / Country<br>Journal. Year [Ref]	Number of patients	Median age, (range)	Age group	Sex (M, F)	Median OS (months)		Prognostic factors
Feng J. et al. / China<br>Human Pathol. 2015 [16]	25	N/A	N/A	N/A	N/A		good: none<br>poor: brainstem
Meyronet D. et al. / France<br>Neuro Oncol. 2017 [33]	21	32,<br>(18-82)	21adults	9, 12	19.6 (n=21)		N/A*
Yoshimoto K. et al. / Japan<br>Brain Tumor Pathol. 2017 [60]	10	15,<br>(5-66)	10(adults and pediatrics)	6, 4	15.7 (n=10)		N/A*
Aboian M.S. et al. / USA<br>AJNR Am J Neuroradiol. 2017 [1]	24	9(mean),<br>(2-19)	24 pediatrics	17, 7	N/A		N/A*
Wang L. et al. / China<br>Human Pathol. 2018 [55]	61	27,<br>(N/A)	26 pediatrics<br>35 adults	13, 13<br>19, 16	13.1 (n=61)		N/A*
Bozkurt SU. et al. / Turkey<br>Childs Nerv Syst. 2018 [7]	31	9.2±3.8,<br>(mean±SD)	31pediatrics	13, 18	N/A		N/A*
Kleinschmidt et al. / USA<br>Clin Neuropathol. 2018 [26]	28	15,<br>(3-81)	15 pediatrics<br>13 adults	7, 7<br>4, 9	6.9 (n=15)<br>8.4 (n=13)		N/A*
Karremann M. et al. / Germany<br>Neuro Oncol. 2018 [25]	62	N/A,<br>(0-18)	56 H3.3, 6 H3.1	30, 31	12.5 (n=61)		N/A*
Ebrahimi A et al. / Germany<br>J Cancer Res Clin Oncol. 2019 [14]	41	29,<br>(4-73)	12 pediatrics<br>29 adults	5, 7<br>19, 10	2.7 (n=7)<br>4.0 (n=19)		N/A*
Karlowee V. et al. / Japan<br>Pathobiology. 2019 [24]	12	30,<br>(6-56)	4 pediatrics<br>8 adults	2, 2<br>6, 2	14.6 (n=12)		good: none<br>poor: *EZH2* expression
Shreck KC. et al. / USA<br>J Neurooncol. 2019 [42]	18	38,<br>(30-68)	18 adults	8, 10	17.6 (n=18)		N/A*
Schulte JD. et al. / USA<br>Neuro Oncol adv. 2020 [44]	60	32,<br>(18-71)	60 adults	33, 27	27.6 (n=60)		good: adults, RT<br>poor: none
Enomoto T et al. / Japan<br>Neurol Med Chir. 2020 [15]	11	N/A	N/A	N/A	17.0 (n=11)		N/A*
Wang Y. et al. / China<br>Front Oncol. 2021 [56]	43	38,<br>(3-75)	13 pediatrics<br>30 adults	6, 7<br>20, 10	12.8 (n=43)		good: preOP KPS ≥ 70, RT<br>poor: *p53* overexpression
Thust S. et al. / UK<br>Quant Imaging Med Surg. 2021 [51]	15	19,<br>(14-64)	15(adults and pediatrics)	6, 9	N/A		not mentioned
Park C. et al. / Korea<br>Cancer Res Treat. 2021 [35]	33	39,<br>(20-70)	33 adults	17, 16	21.8 (n=33)		not identified
Schuller U. et al. / Germany<br>Acta Neuropathol. 2021 [43]	83	17(n=78),<br>(3-58)	83(adults and pediatrics)	45, 36 (n=81)	9.0 (n=63)		good: adults, supratentrial locations, *FGFR1* mutation<br>poor: *TP53* mutation
Zhao et al. / China<br>BMC Neurol. 2022 [61]	14	19,<br>(8-70)	14(adults and pediatrics)	11, 3	16.8 (mean)		N/A*
Zheng L. et al. / China<br>Am J Surg Pathol. 2022 [62]	164	23,<br>(3-71)	70 pediatrics<br>94 adults	36, 34<br>52, 42	5.0 (n=N/A)<br>16.0 (n=N/A)	mOS 10.5<br>(n=122)	good: tumor diameter ≤ 3cm, Ki-67 ≤ 5%<br>poor: pediatrics, brainstem, *ATRX* expression
Jang SW. et al. / Korea<br>Brain Tumor Res Treat. 2022 [23]	24	24(mean),<br>(4-73)	11 pediatrics<br>13 adults	6, 5<br>6, 7	10.4 (n=24)		not identified
Present study	93	31,<br>(4-78)	26 pediatrics<br>67 adults	18, 8<br>37, 30	15.3 (n=24)<br>16.7 (n=63)	mOS 16.6<br>(n=87)	good: female sex, pre OP KPS ≥ 80, RT ≥ 50Gy<br>poor: none

N/A: Not Available, * compaired with H3 wild type
