# Supplementary material for: Neuroradiological, genetic and clinical characteristics of histone H3 K27-mutant diffuse midline gliomas in the Kansai Molecular Diagnosis Network for CNS Tumors (Kansai Network): multicenter retrospective cohort
Source: Acta Neuropathol Commun. 2024 Jul 27;12:120. doi: 10.1186/s40478-024-01808-w (PMC11282756; doi:10.1186/s40478-024-01808-w)
Supplement: Supplementary file 3 — Additional file 3: Table S3. Discordance between imaging features (low/high grade) and histological findings (presence or absence of GBM features) in the biopsy cases. [file 40478_2024_1808_MOESM3_ESM.pdf]

Supplementary Table 3

Table S3 Discordance between imaging features (low/high grade) and histological findings (presence or absence of GBM features) in the biopsy cases

Biopsy cases	Imaging features (High or Low grade)*									
	Total (n = 53)		Thalamus (n = 22)		Brainstem (n = 19)		Spinal cord (n = 6)		Others (n = 6)	
	High (n = 37)	Low (n = 16)	High (n = 17)	Low (n = 5)	High (n = 9)	Low (n = 10)	High (n = 6)	Low (n = 0)	High (n = 5)	Low (n = 1)
**Histology (GBM features, Presence or Absence)†**										
(+) (n = 14)	13 (25%)	1 (2%)	6 (27%)	1 (5%)	3 (16%)	0 (-)	2 (33%)	0 (-)	2 (33%)	0 (-)
(-) (n = 39)	24 (45%)	15 (28%)	11 (50%)	4 (18%)	6 (31%)	10 (53%)	4 (67%)	0 (-)	3 (50%)	1 (17%)

* Gd-enhanced images were not available in 5 cases (3 Thalamus, 1 Spinal cord and 1 Others)

† Histology was unknown in one case (Spinal cord)
