# Supplementary material for: Neuroradiological, genetic and clinical characteristics of histone H3 K27-mutant diffuse midline gliomas in the Kansai Molecular Diagnosis Network for CNS Tumors (Kansai Network): multicenter retrospective cohort
Source: Acta Neuropathol Commun. 2024 Jul 27;12:120. doi: 10.1186/s40478-024-01808-w (PMC11282756; doi:10.1186/s40478-024-01808-w)

Supplementary Figure 1

MR imaging of histone H3 K27-mutant gliomas in Kansai Network

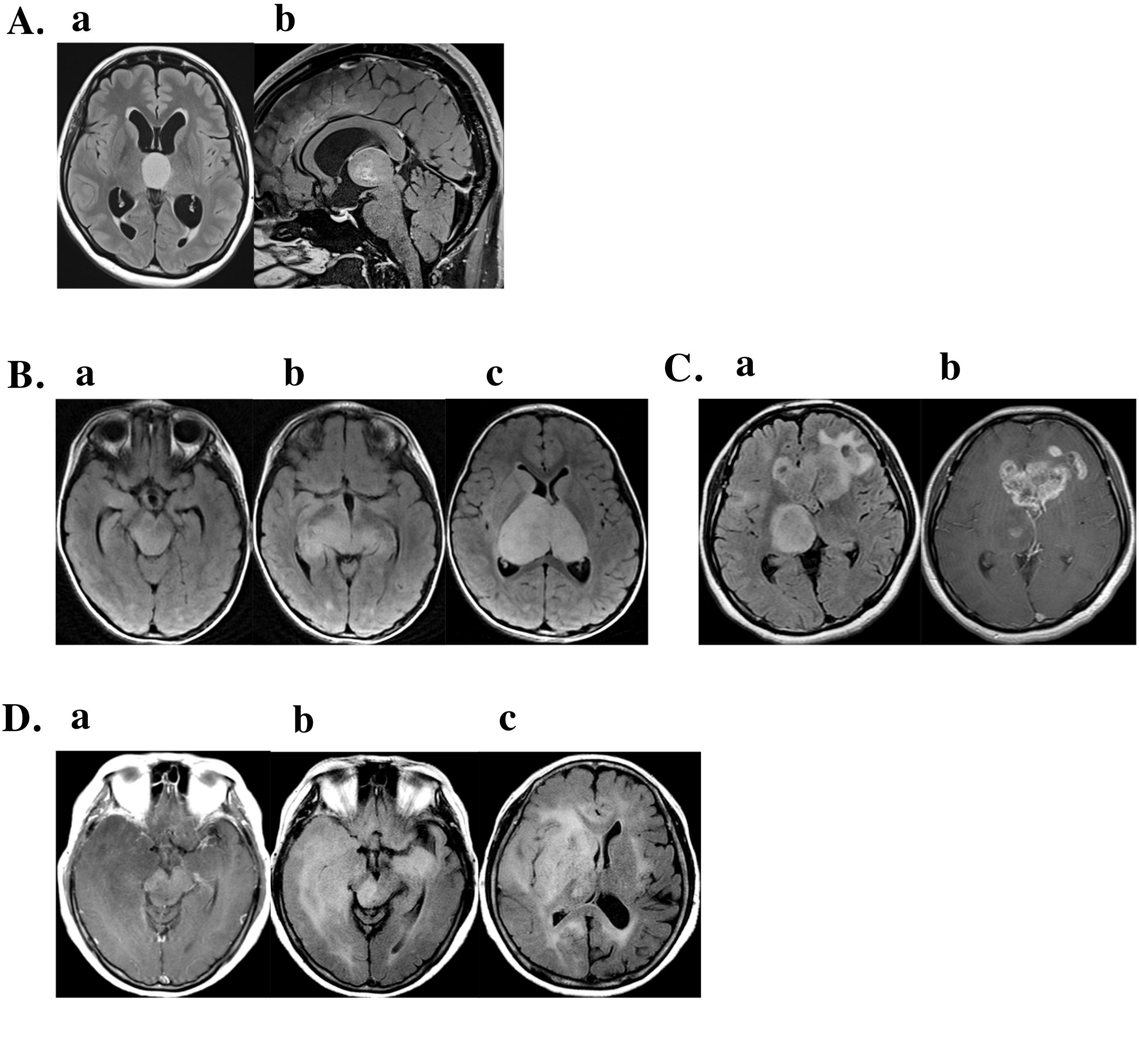

Supplement: Supplementary file 4 — Additional file 4: Figure S1. MR imaging of histone H3 K27-mutant gliomas in Kansai Network. A. a, b: FLAIR, The tumor is a sole lesion, and main location is the third ventricle: Others/midline (included). B. a, b, c: FLAIR, The tumor is comprised of contiguous multifocal lesions, and main location is the thalamus: Thalamus/midline (included). C. a: FLAIR, b:T1-Gd, The tumor is comprised of non-contiguous multifocal lesions, and the main location is the thalamus and/or corpus callosum, unclassified tumor: Others/midline (included). D. a: T1-Gd, b, c: FLAIR, The tumor is comprised of non-contiguous multifocal lesions, and the main location is unclassified, the cerebral hemisphere is more involved than the brainstem : Others/non-midline (excluded). E. a, b: FLAIR, c:T1-Gd, The tumor is comprised of contiguous multifocal lesions, and the main location is the left basal ganglia, which involve the thalamus and/or the brainstem more than the cerebral hemisphere: Others/midline (included). F. a: FLAIR, b:T1-Gd, The tumor is comprised of contiguous multifocal lesions, and the main location is the right medial temporal lobe: Cerebral hemisphere/non-midline (excluded) [file 40478_2024_1808_MOESM4_ESM.pdf]
