# Supplementary material for: Neuroradiological, genetic and clinical characteristics of histone H3 K27-mutant diffuse midline gliomas in the Kansai Molecular Diagnosis Network for CNS Tumors (Kansai Network): multicenter retrospective cohort
Source: Acta Neuropathol Commun. 2024 Jul 27;12:120. doi: 10.1186/s40478-024-01808-w (PMC11282756; doi:10.1186/s40478-024-01808-w)

Supplementary Figure 2

Kaplan–Meier survival curves according to clinical factors:

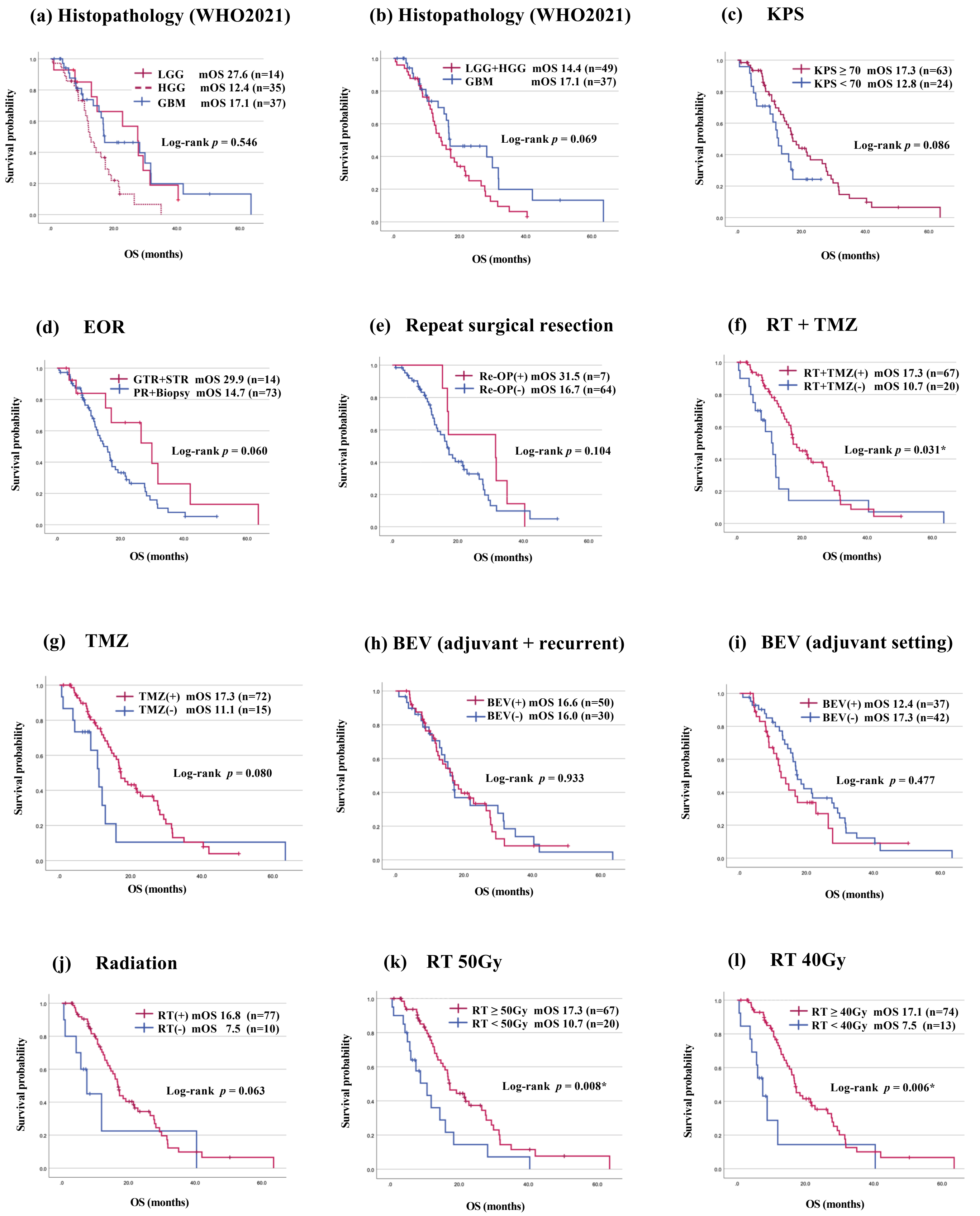

Supplement: Supplementary file 5 — Additional file 5: Figure S2. Kaplan–Meier survival curves according to clinical factors: histopathology (LGG vs. HGG without GBM features vs. GBM features) (a), histopathology (LGG + HGG without GBM features vs. GBM features) (b), preoperative KPS score (≥ 70 vs. < 70) (c), EOR (GTR + STR vs. PR + Biopsy) (d), repeat surgical resection (e), RT+TMZ (f), TMZ (g), BEV (adjuvant + recurrent) (h), BEV (adjuvant) (i), Radiation (j), RT (≥ 50 Gy vs. < 50 Gy) (k), RT (≥ 40 Gy vs. < 40 Gy) (l). [file 40478_2024_1808_MOESM5_ESM.pdf]
