# Supplementary figures and images for: Neuroradiological, genetic and clinical characteristics of histone H3 K27-mutant diffuse midline gliomas in the Kansai Molecular Diagnosis Network for CNS Tumors (Kansai Network): multicenter retrospective cohort
Source: Acta Neuropathol Commun. 2024 Jul 27;12:120. doi: 10.1186/s40478-024-01808-w (PMC11282756; doi:10.1186/s40478-024-01808-w)

# Supplementary Figure 3

Kaplan–Meier survival curves of thalamus

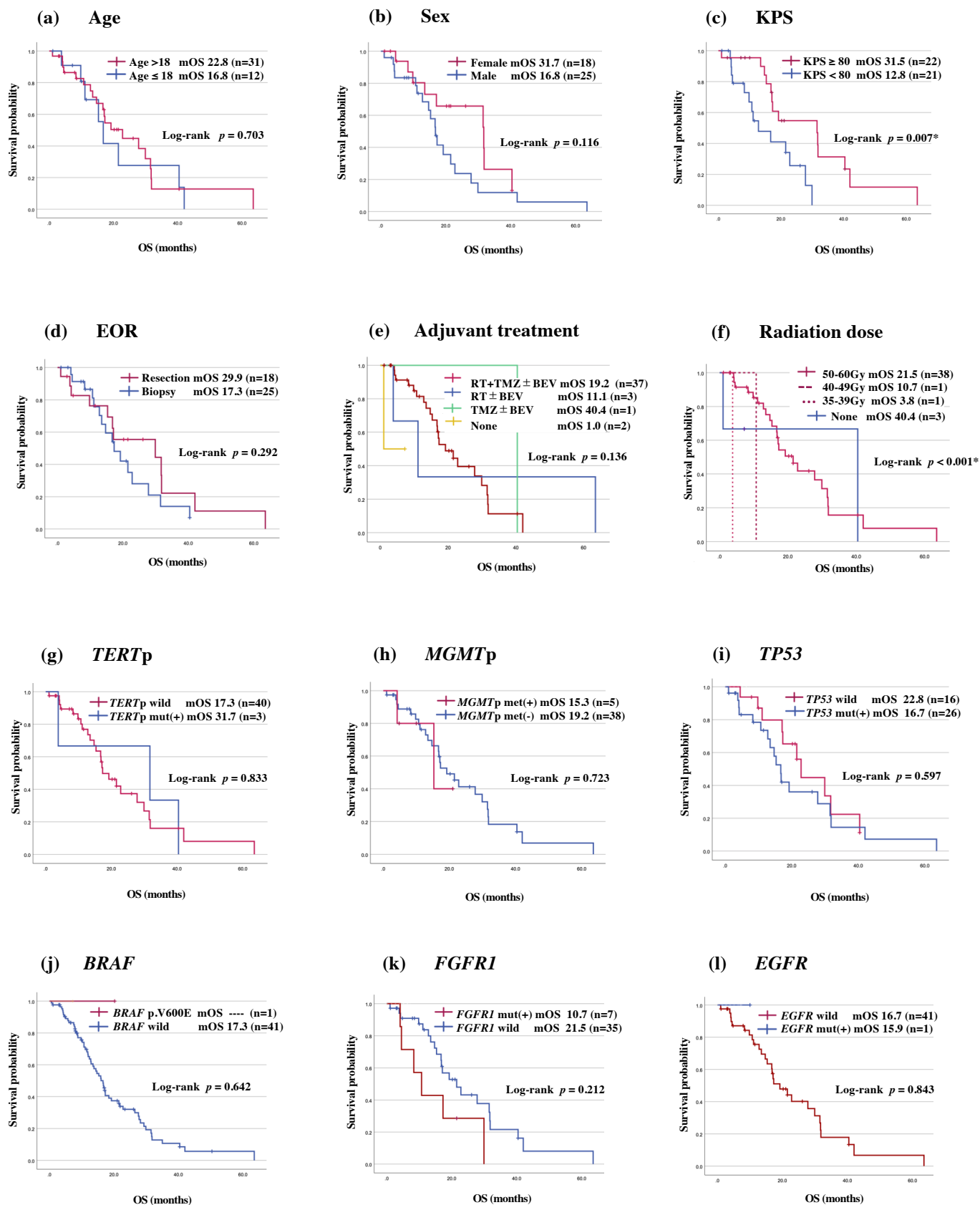

Supplement: Supplementary file 6 — Additional file 6: Figure S3. (Thalamus). Kaplan–Meier survival curves according to clinical factors: age (a), sex (b), preoperative KPS score (c), extent of surgical resection (d) adjuvant treatment (e) and radiation dose (f), molecular factors: TERT (g), MGMT (h), TP53 (i), BRAF (j), FGFR1 (k) and EGFR (l) in the study cohort. [file 40478_2024_1808_MOESM6_ESM.pdf]

# Supplementary Figure 4

Kaplan–Meier survival curves of brainstem

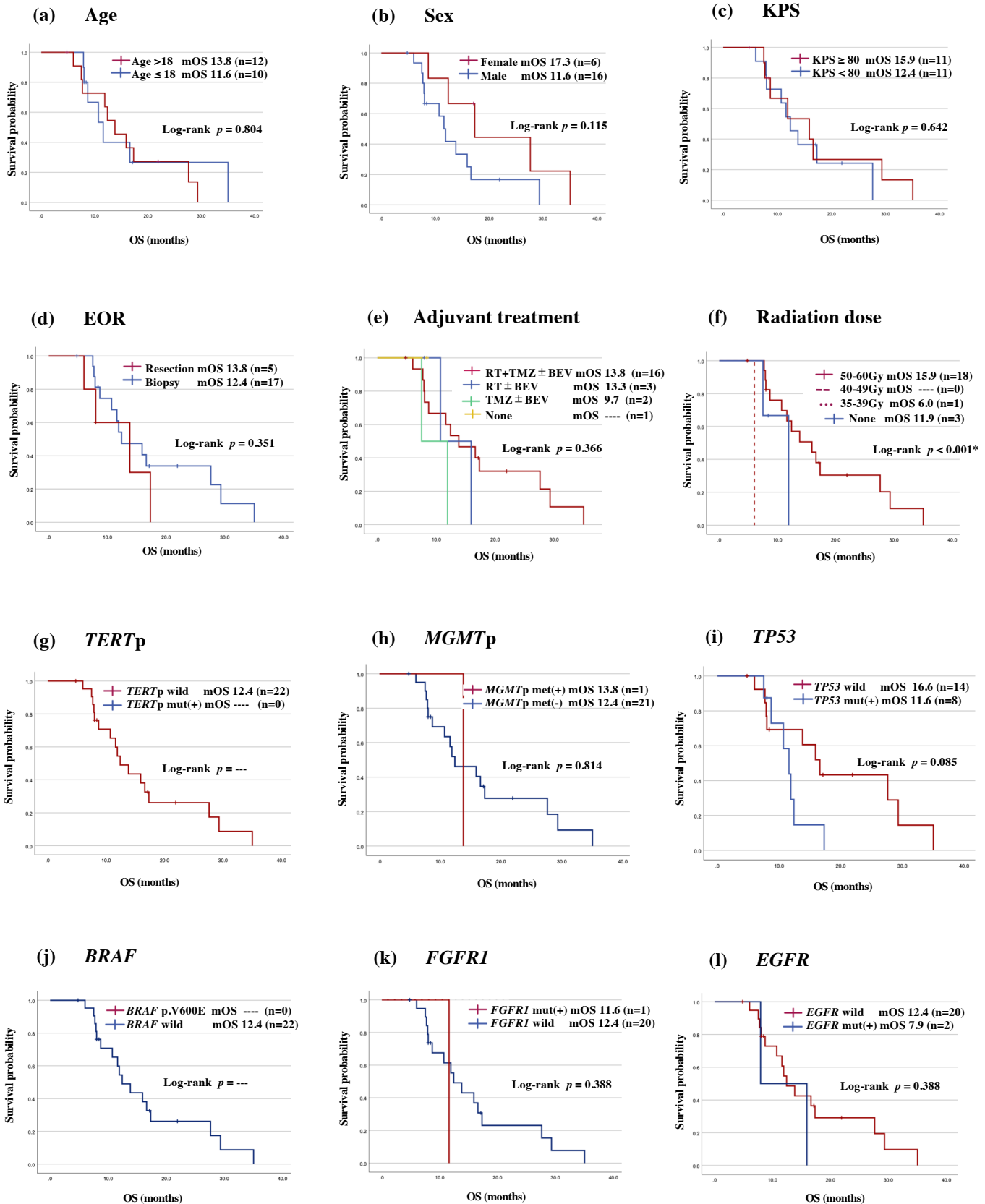

Supplement: Supplementary file 7 — Additional file 7: Figure S4. (Brainstem). Kaplan–Meier survival curves according to clinical factors: age (a), sex (b), preoperative KPS score (c), extent of surgical resection (d) adjuvant treatment (e) and radiation dose (f), molecular factors: TERT (g), MGMT (h), TP53 (i), BRAF (j), FGFR1 (k) and EGFR (l) in the study cohort. [file 40478_2024_1808_MOESM7_ESM.pdf]

# Supplementary Figure 5

Kaplan–Meier survival curves of spinal cord

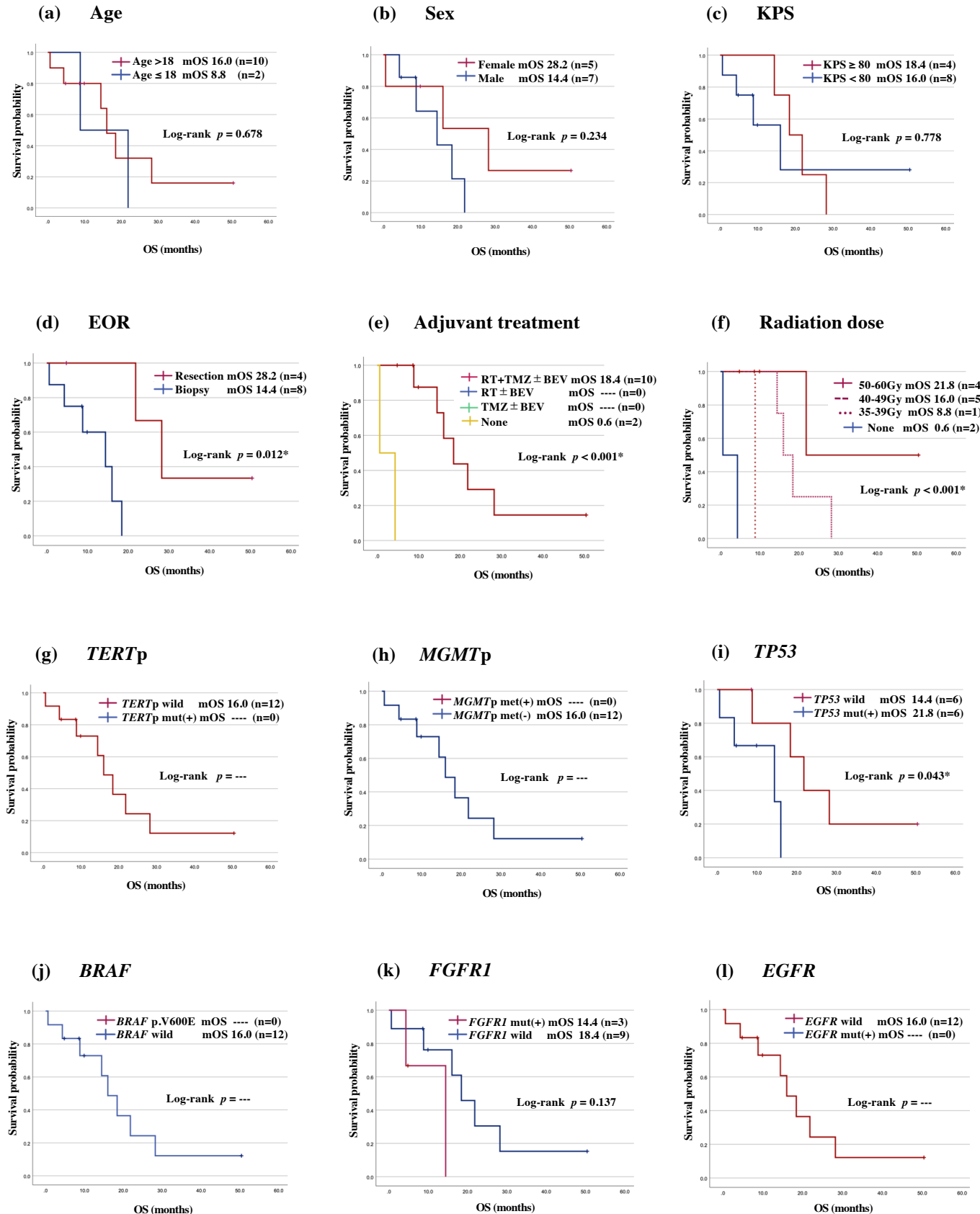

Supplement: Supplementary file 8 — Additional file 8: Figure S5. (Spinal cord). Kaplan–Meier survival curves according to clinical factors: age (a), sex (b), preoperative KPS score (c), extent of surgical resection (d) adjuvant treatment (e) and radiation dose (f), molecular factors: TERT (g), MGMT (h), TP53 (i), BRAF (j), FGFR1 (k) and EGFR (l) in the study cohort. [file 40478_2024_1808_MOESM8_ESM.pdf]

# Supplementary Figure 6

Kaplan–Meier survival curves of other midline locations

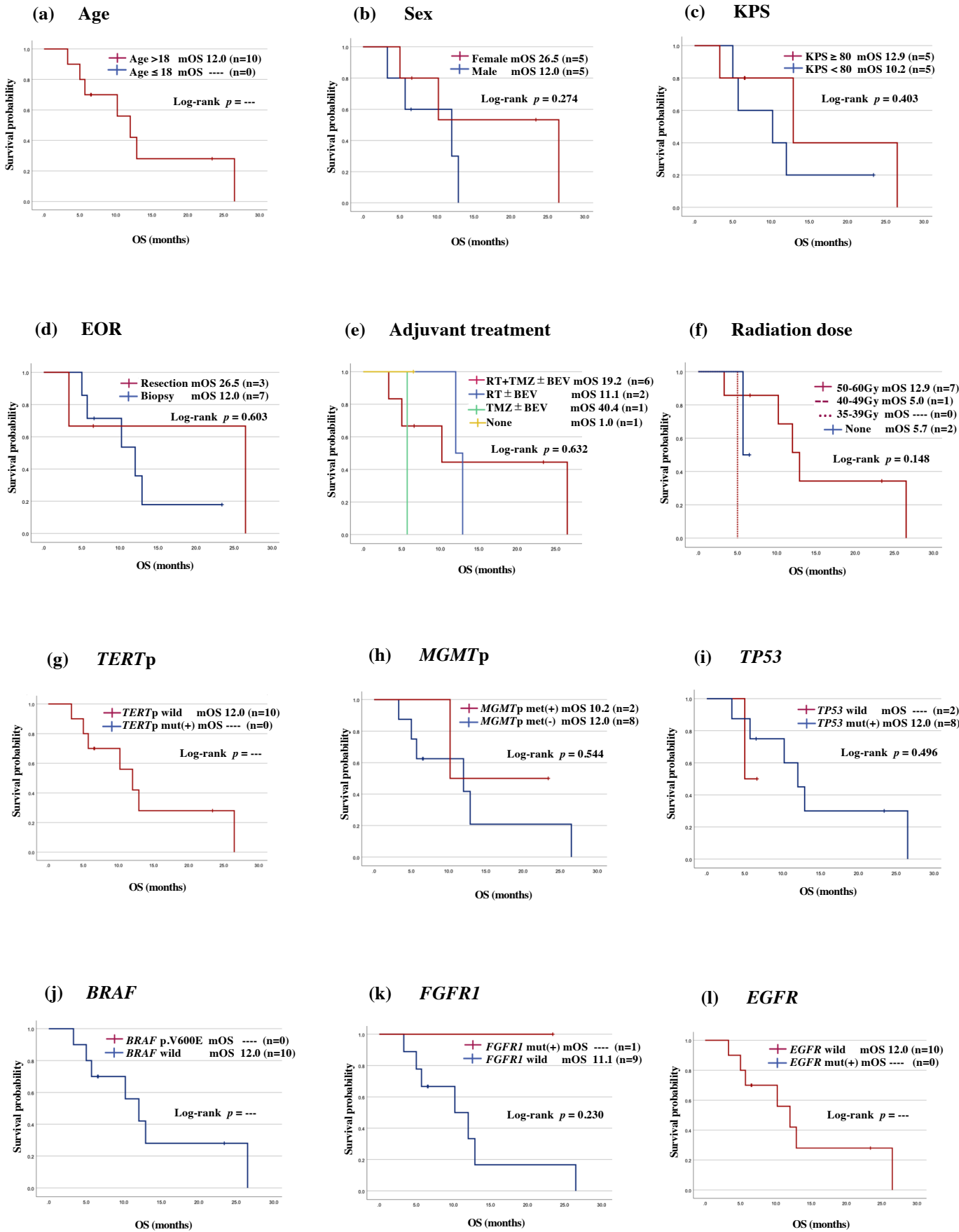

Supplement: Supplementary file 9 — Additional file 9: Figure S6. (Other midline location). Kaplan–Meier survival curves according to clinical factors: age (a), sex (b), preoperative KPS score (c), extent of surgical resection (d) adjuvant treatment (e) and radiation dose (f), molecular factors: TERT (g), MGMT (h), TP53 (i), BRAF (j), FGFR1 (k) and EGFR (l) in the study cohort. [file 40478_2024_1808_MOESM9_ESM.pdf]
