# Supplementary material for: Neuroradiological, genetic and clinical characteristics of histone H3 K27-mutant diffuse midline gliomas in the Kansai Molecular Diagnosis Network for CNS Tumors (Kansai Network): multicenter retrospective cohort
Source: Acta Neuropathol Commun. 2024 Jul 27;12:120. doi: 10.1186/s40478-024-01808-w (PMC11282756; doi:10.1186/s40478-024-01808-w)

Supplementary Figure 7

Kaplan–Meier survival curves according to radiation dose without spinal cord group

(a) Radiation dose
(w/o spinal cord group)

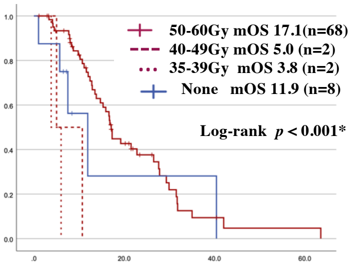

(b) RT 50Gy
(w/o spinal cord group)

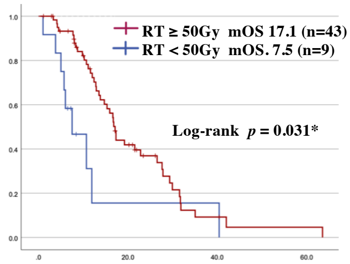

(c) RT 40Gy
(w/o spinal cord group)

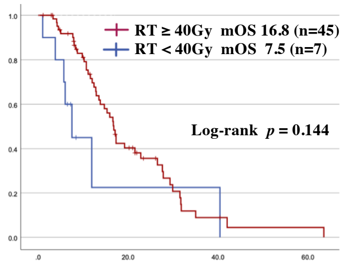

Supplement: Supplementary file 10 — Additional file 10: Figure S7. Kaplan–Meier survival curves according to radiation dose without spinal cord group: Radiation dose (a), RT (≥ 50 Gy vs. < 50 Gy) (b), RT (≥ 40 Gy vs. < 40 Gy) (c) [file 40478_2024_1808_MOESM10_ESM.pdf]
